# Nanomedicine for targeting cancer-associated fibroblasts in cancer therapy

**DOI:** 10.7150/thno.120283

**Published:** 2026-01-01

**Authors:** Zi-Yi Chen, Han-Zhe Liu, Zheng-Jun Shang, Guo-Feng Luo, Xian-Zheng Zhang

**Affiliations:** 1 State Key Laboratory of Oral & Maxillofacial Reconstruction and Regeneration, Key Laboratory of Oral Biomedicine Ministry of Education, Hubei Key Laboratory of Stomatology, School & Hospital of Stomatology, Wuhan University, Wuhan 430079, P. R. China; 2 Taikang Center for Life and Medical Sciences of Wuhan University, Wuhan University, Wuhan 430079, P. R. China; 3 Key Laboratory of Biomedical Polymers of Ministry of Education & Department of Chemistry, Wuhan University, Wuhan 430072, P. R. China

**Keywords:** cancer-associated fibroblasts, nanomedicine, tumor microenvironment, drug delivery, cancer therapy

## Abstract

Cancer-associated fibroblasts (CAFs) play a crucial role in the tumor microenvironment, where they facilitate tumor progression, angiogenesis, immune evasion, and treatment resistance, highlighting the urgent need for CAF-targeted strategies for high-performance tumor therapy. Recent nanomedicine approaches have shown promise in CAFs targeting in order to achieve precise targeting, spatiotemporal control of drug release, and enhanced drug penetration into dense fibrotic stroma. Accordingly, this review summarizes emerging nanotechnologies that address challenges through the development of functional nanomaterials for CAFs targeting, including polymers, metal and non-metal inorganic nanoparticles (NPs), cell membrane-based NPs, and protein-based NPs. Specifically, various therapeutic approaches such as direct CAFs depletion, signaling pathway modulation in CAFs, and CAFs reprogramming by using these nanomedicines are discussed. Furthermore, potential avenues for future studies, including the development of versatile nanosystems and the exploration of personalized treatment regimens, and challenges of advanced functional nanomaterials are involved as well. We hope that this review will offer new insights into cancer therapy and advance the development of clinically applicable CAF-targeted nanomedicines.

## 1. Introduction

A solid tumor can be analogized to a complex architectural structure, comprising primarily tumor cells, stromal cells, and the extracellular matrix (ECM). Together, these constituents create a highly intricate tumor microenvironment (TME) that supports tumor development and progression. Amongst these, CAFs act as the principal orchestrators, performing multifaceted roles such as synthesizing and remodeling the ECM, as well as secreting a variety of cytokines or metabolic factors that facilitate tumor progression, invasion, and metastasis 1. Furthermore, CAFs regulate tumor immune suppression and promote angiogenesis 2. For instance, in human breast carcinomas, Orimo et al. revealed that resident CAFs overexpressed stromal cell-derived factor 1 (SDF-1), simultaneously recruiting endothelial progenitor cells to accelerate angiogenesis and directly stimulating carcinoma proliferation via C-X-C chemokine receptor type 4 (CXCR4) 3. Furthermore, Shintani et al. demonstrated that non-small cell lung cancer-associated CAFs released interleukin 6 (IL-6), which synergized with transforming growth factor *β* (TGF-*β*) to promote epithelial-mesenchymal transition (EMT) and conferred cisplatin resistance, thereby illustrating the critical role of CAF-derived cytokines in dictating therapeutic outcomes 4. Moreover, CAFs have also been identified as the main contributor to establishing physical and biochemical barriers that impede the penetration of therapeutic agents and/or immune cells, thereby reducing treatment efficacy 5, 6. Collectively, these roles indicate CAFs as critical stromal targets for tumor therapy. However, the heterogeneity and plasticity of CAFs increase the complexity of treatment 7. Some CAF-directed therapies have failed to achieve desired outcomes or effectively modulate CAFs activity, and in certain cases, have even inadvertently accelerated disease progression 8. Notably, CAFs exhibit phenotypic reversibility and comprise highly diverse subpopulations 9. Their functional plasticity in response to environmental signals suggests the potential of spatiotemporally controlled targeting strategies to improve treatment efficacy 10. Thus, advances in identifying distinct CAFs subsets have not only enriched our understanding of their phenotypic and functional diversity, but also emphasized the need for delivery platforms with improved specificity.

In recent years, the pursuit of more effective cancer therapies has driven rapid progress in nanomedicine, a field that continues to advance therapeutic innovation 11. NPs ranging from 50-300 nm, including metal, polymer, monoclonal antibody (mAb) and extracellular vesicle (EVs)-based systems, have shown great potential as versatile drug delivery platforms 12-17. Unlike conventional molecular drugs, nanomaterials possess distinct physicochemical properties derived from their submicroscopic size, morphology, structure, and surface functionality 18-20. Their capacity to efficiently encapsulate therapeutic agents and achieve enhanced tumor accumulation via mechanisms such as the enhanced permeability and retention (EPR) effect has been widely documented 21. Moreover, their exceptional modularity allows for precise engineered NPs to actively target tumors and release drugs under specific conditions within the TME, such as acidic pH, tumor-associated enzymes, as well as external stimuli (e.g., light, ultrasound, magnetic fields and temperature) 22-25. More importantly, multiple drugs can be co-loaded within a single nanoplatform for combination therapy, which can significantly improve treatment efficacy while reducing systemic side effects 26.

Based on these advantages, the dynamic bidirectional interaction between CAFs and engineered nanomaterials has attracted research interest. Targeting CAFs with nanomaterials represents a promising avenue for enhancing cancer treatment 27-29. It is worth emphasizing that although CAF-directed interventions can remodel the TME and improve drug penetration, their full clinical potential will likely be realized through rational design of dual-targeting systems that simultaneously eliminate cancer cells and disrupt CAF-mediated pro-tumorigenic functions. Cooperative interactions between chemotherapy, radiotherapy, immunotherapy, photodynamic therapy (PDT), photothermal therapy (PTT) or magnetic hyperthermia, can produce synergistic effects when they are spatially and temporally orchestrated via a single nanoplatform 30, 31. Given the critical role of CAFs in tumorigenesis and progression, along with the considerable potential of NPs-based systems designed to target CAFs, this review provides a comprehensive overview of CAF-targeted nanostrategies for cancer treatment. To this end, we firstly summarize the origin, differentiation, and biomarkers of CAFs, as well as their interactions with cancer and stromal cells. On the basis of this, a comprehensive overview of cutting-edge NPs (e.g., polymers, metal and non-metal inorganic NPs, cell membrane-based NPs, and protein-based NPs) used for CAFs targeting will be presented. Strategies, such as direct CAFs depletion, targeting specific signaling/metabolic pathways in CAFs, and engineering CAFs, are highlighted as well, and representative studies throughout the following sections will deliberately interweave how these nanoplatforms concurrently deliver conventional anti-cancer modalities and CAF-targeting agents to achieve multimodal synergistic effects. Finally, we discuss ongoing challenges and propose future research directions with clinical translational potential will be discussed, offering new perspectives for advancing cancer treatment.

## 2. Characteristics of cancer-associated fibroblasts and their function in tumor ecology

**Figure 1:**
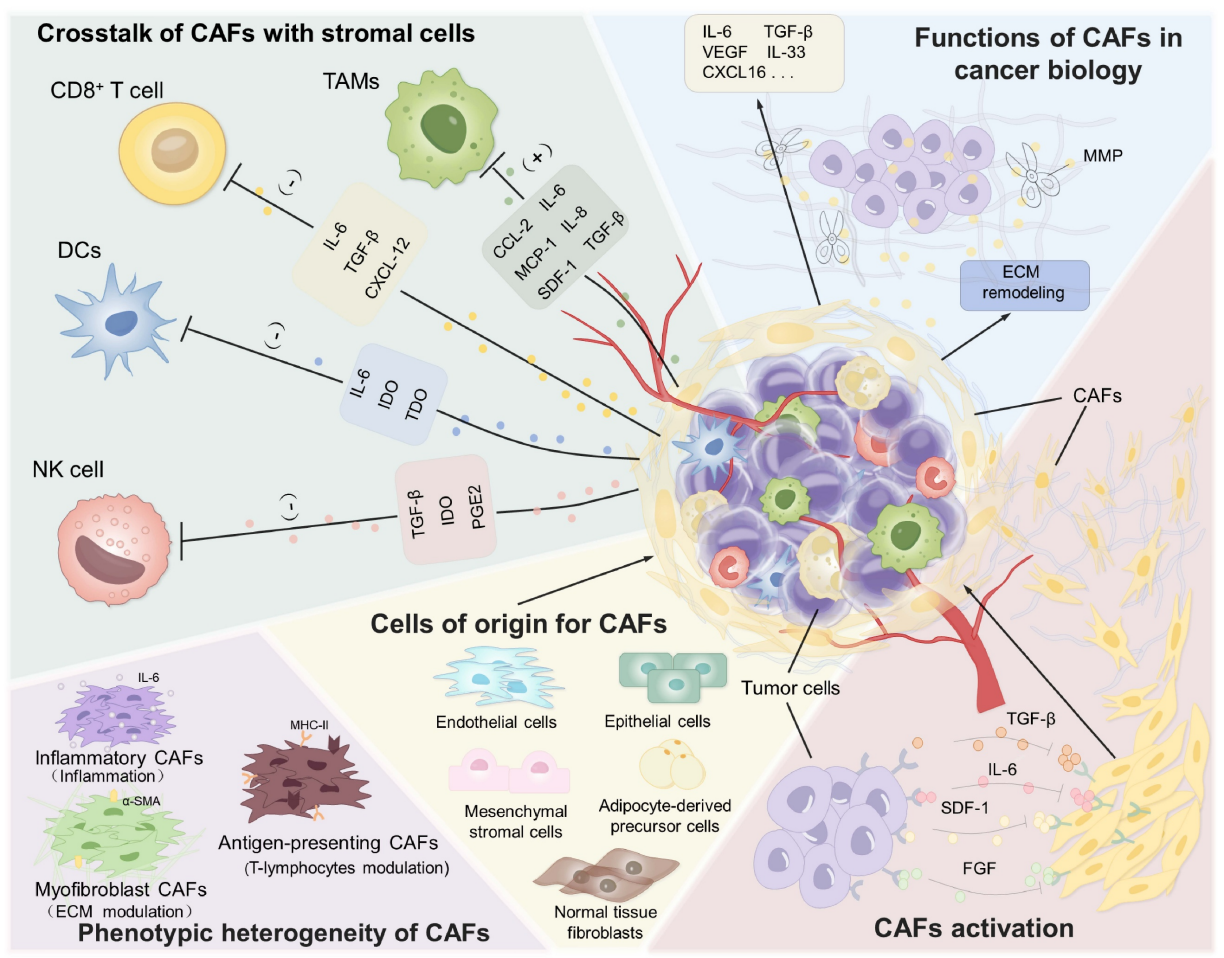
Characterization and function of CAFs in TME. CAFs reveal their importance through multiple functions such as interaction with various types of stromal cells, multiple pathways of origin, and involvement in tumor growth and metastasis.

CAFs represent a fundamental cellular constituent within tumors, playing an important role in cancer progression, invasion, metastasis, and immune evasion (**Figure 1**). They secrete a range of cytokines and metabolites that promote tumor growth and metastasis 32-34. Moreover, as the primary source of ECM components, CAFs contribute to the formation of a dense physical and biochemical barrier that restricts the efficient penetration of therapeutic agents and immune cells, thereby significantly diminishing treatment efficacy 35, 36. Enhancing our understanding of CAFs biology will shed light on their dynamic complexity and functional plasticity within the TME, thereby facilitating the rational design of more effective CAF-targeting strategies.

### 2.1. The origin, biomarkers and heterogeneity of CAFs

CAFs are a distinct population of activated fibroblasts found in various tumor types. Extensive research has established their high heterogeneity in phenotype, origin, and function, reflecting their multifaceted roles within the TME. This diversity may be attributed to their derivation from multiple cellular sources and differentiation pathways 37-39. For instance, normal tissue fibroblasts can be reprogrammed into CAFs in response to factors secreted by cancer cells. As demonstrated by Procopio et al., keratinocyte-derived fibroblast growth factor-2 (FGF-2) played a critical role in down-modulating *p53* and CSL in adjacent dermal fibroblasts, thereby relieving repression of senescence-escape and CAFs effector genes including *α*-smooth muscle actin (*α*-SMA), periostin and cyclooxygenase-2 and facilitating the rapid conversion of quiescent fibroblasts into fully activated CAFs within premalignant and malignant skin lesions 40. Cellular origins of CAFs include adipocyte-derived precursor cells, mesenchymal stromal cells recruited from bone marrow, endothelial cells through EMT, pericytes or epithelial cells. After their initial conversion, CAFs expansion and sustained activation are mainly driven by tumor-secreted factors such as TGF-*β*, PDGF, and IL-6 41-44. Recent technological advances in biomarker profiling and single-cell sequencing have resolved CAFs into distinct subpopulations that can be traced from normal tissue to advanced tumors 45-48. For example, in murine pancreatic ductal adenocarcinoma, the three resident fibroblast pools (FB1, FB2, and FB3) remodel along different paths during tumorigenesis: FB1/2 converge into inflammatory CAFs (iCAFs) rich in IL-6, IL-11 and other immunosuppressive cytokines, whereas FB3 evolve into *α*-SMA-high myofibroblastic CAFs (myCAFs) that lay down a stiff, drug-blocking extracellular matrix 49. This dynamic iCAFs-myCAFs spectrum illustrates how tumor-derived cues actively reprogram CAFs identity and function to fuel tumor growth.

This diversity of origins is further reflected in the molecular markers associated with CAFs. Widely recognized markers include *α*-SMA, fibroblast activation protein (FAP), fibroblast-specific protein 1, and platelet-derived growth factor receptor-*β* (PDGFR-*β*) 50-52. Among these, certain markers exhibit unique features that make them ideal targets for nanomedicine-based CAF-directed therapies. *α*-SMA is a key indicator of activated fibroblasts and is commonly used to evaluate the outcome of CAF-targeting therapies 53, 54. However, its expression in non-cancerous myofibroblasts (e.g., in wound healing) may reduce targeting specificity 55. Another important marker, FAP, is selectively expressed by CAFs in most human epithelial cancers and in reactive stroma during chronic inflammation 56. Notably, Yang et al. demonstrated that FAP expression defined a distinct subset of iCAFs that drove immunosuppression through signal transducer and activator of transcription 3 (STAT3)-dependent up-regulation of c-c motif chemokine ligand 2 (CCL2), reinforcing its relevance as a CAF-specific target 57. As emphasized in recent studies, optimal ligand density and presentation are critical for successful stromal targeting, guiding the design of FAP-directed nanocarriers that exhibit enhanced tumor accumulation 58, 59. Additionally, proteins such as Asporin, microfibril-associated protein 5, and collagen 11-*α*1 are often exclusively expressed in CAFs, distinguishing them from other cell types 60, 61. Crucially, passive accumulation based solely on the EPR effect cannot exploit these differences: a quantitative meta-analysis indicated that only 0.7% of systemically injected NPs reached solid tumors in humans 62. Consequently, the integration of CAF-restricted ligands into nanocarriers with active targeting property is essential for reliable and highly specific delivery. Identifying and characterizing proteins exclusively expressed in CAFs could enhance their potential as precise targets in cancer therapy.

However, accumulating evidence demonstrates that CAFs subsets with the same hallmark can also exhibit tumor-restraining properties. For instance, global genetic depletion of all *α*-SMA^+^ CAFs in a murine PDAC model unexpectedly accelerated tumor growth and reduced survival, indicating that indiscriminate elimination of this compartment may remove protective functions 63. Similarly, antigen-presenting CAFs (apCAFs) characterized by high MHC-II expression and preferential localization near tertiary lymphoid structures in gastric cancer, have been shown to activate T cells and enhance cytotoxic function in preclinical models 64. Together, these findings emphasize the need for strategies that discriminate between pro- and anti-tumor subsets rather than broad CAFs depletion while exploiting CAFs plasticity for therapeutic benefit. Recognizing that CAFs comprise both tumor-promoting and tumor-restraining populations, the next step is to deploy high-resolution tools and targeted interventions that neutralize the harmful subsets while preserving or enhancing protective ones. Single-cell spatial multi-omics recently applied to over 14 million cells across ten cancer types-have identified four spatially organized CAFs subtypes in human and murine pancreatic tumors, providing the detailed insights necessary for such distinctions 65. Moreover, environmental cues can actively redirect CAFs toward an anti-tumor state. For example, oral administration of Bifidobacterium adolescents enriched CD143^+^ CAFs that expressed the tumor suppressor growth arrest specific 1 (*GAS1*) and significantly inhibited colorectal tumor growth 66. By integrating high-resolution molecular mapping with functional assays, next-generation therapies can selectively target pro-tumorigenic CAFs, preserve or augment tumor-restraining populations, and ultimately improve patient outcomes.

### 2.2 Crosstalk of CAFs in tumor microenvironment

For progressive tumor development, there is a dynamic reciprocal crosstalk between tumor cells and CAFs 67. On one hand, cancer cells secrete a spectrum of cytokines and growth factors that convert quiescent fibroblasts into activated CAFs, effectively recruiting the stroma to facilitate tumor growth 68. For example, TGF-*β* produced by tumor cells plays a critical role in promoting fibrosis by activating fibroblasts through both Drosophila mothers against decapentaplegic (Smad) and non-Smad pathways 69, 70. Other factors, including PDGF, FGF, IL-6 and interleukin 11 (IL-11), govern the activation process as well 71-74. Notably, metabolites like lactate released by cancer cells can also activate CAFs through hypoxia-inducible factor-1*α* (HIF-1*α*) signaling, driving the pro-tumorigenic phenotype conversion of CAFs 75. Conversely, CAFs exhibit enhanced glycolytic flux and promoted secretion of lactate, which can be taken up by tumor cells to fuel their invasion and metastasis 76. Besides, cancer cell-derived exosomes, carrying proteins, mRNAs, and miRNAs, also play a key role in modulating CAF function and establishing a self-reinforcing tumor-promoting ecosystem 77, 78. Additionally, contact-dependent interactions mediated by integrin *β*1/focal adhesion kinase (FAK) and N-cadherin ligation transmits tension-dependent signals from cancer cells to CAFs, leading to enhanced CAFs proliferation and matrix-invasive capacity 79.

On the other hand, CAFs actively facilitate tumor growth, proliferation, and metastasis. CAFs secrete abundant ECM components (e.g., collagens, hyaluronic acid, and tenascin C, cross-linking collagen I, matrix metalloproteinases (MMPs)) to provide a favorable microenvironment for tumor growth 80-83. They also secrete various growth factors and cytokines, such as IL-6, interleukin 33 (IL-33) and C-X-C motif chemokine ligand 16 (CXCL16), to promote cancer cell proliferation and survival 84-87. Furthermore, CAFs also alter the TME through the secretion of MMPs and other ECM-remodeling enzymes that direct tumorigenesis and progression 88. By producing and linearizing collagen fibers, CAFs form a physical barrier that impedes drug penetration and compromises treatment efficacy 89. Moreover, CAFs orchestrate therapeutic resistance via complex signaling networks, positioning CAF-targeted therapies as a promising strategy to overcome drug resistance 90. Recent studies have identified constitutive activation of the Yes-associated protein (*YAP*) as a hallmark of CAFs. *YAP* regulated the expression of cytoskeletal regulators such as anillin (*ANLN*), diaphanous-related formin-3 (*DIAPH3*), and myosin light chain 9 (*MYL9*). Matrix stiffness further amplified *YAP* activation, establishing a self-reinforcing loop that sustained CAFs activity 91. CAFs also promote angiogenesis by secreting vascular endothelial growth factor (VEGF) and other pro-angiogenic factors, thereby enhancing oxygen and nutrient supply to the tumor 92, 93. CAFs additionally influence cancer cell metabolism 94. For example, they enhance glycolysis and adenosine triphosphate (ATP) production in ovarian cancer cells, supporting their proliferation 95. CAF-derived EVs also contribute to tumor progression by promoting migration, invasion, and ECM remodeling in oral squamous cell carcinoma (OSCC) 96. These EVs can communicate with distant organs to form pre-metastatic niches, as demonstrated in lung fibroblasts transformed by CAF-derived TGF-*β* signaling 97. Overall, CAFs engage in extensive crosstalk with cancer cells to collectively promote tumor growth and therapy resistance.

Beyond direct interactions with cancer cells, CAFs collaborate with other stromal cells to establish an inflammatory, pro-angiogenic, and immunosuppressive TME 98. They recruit pro-tumorigenic myeloid cells, such as myeloid-derived suppressor cells, to facilitate invasion, angiogenesis, and suppression of adaptive immunity 99, 100. CAFs also contribute to immune evasion by excluding cytotoxic T cells from tumor nests 101. A recent study in head and neck squamous cell carcinoma identified a CAF subset expressing high levels of CXCL9/CXCL10/CXCL12 and major histocompatibility complex class I (MHC I) molecules, which played critical role in limiting CD8^+^ T cell infiltration and promoted T cell dysfunction 102. In addition to immune modulation, CAFs enhance angiogenesis by recruiting endothelial progenitor cells via SDF-1 103. In conclusion, CAFs play a central role in TME to drive tumor progression by producing abundant ECM and mediate multicellular communication through cytokines, EVs and matrix signals.

### 2.3 Therapeutic benefits of CAFs disruption

Given the important role of CAFs in tumor progression, targeted disruption of CAFs offers substantial therapeutic benefits, including 1) restoring anti-tumor immunity by alleviating CAF-mediated immune exclusion; 2) blocking CAF-activated oncogenic signaling; 3) disrupting CAF-driven matrix stiffening and metastatic niche formation. For instance, selective depletion of FAP-*α*^+^ CAFs enhanced anti-cancer immunity by increasing CD8^+^ T cell infiltration, resulting in significant suppression of primary tumor growth 104. In pancreatic cancer, inactivation of CAFs disrupted stromal TGF-*β* signaling, leading to reduced TGF-*β* secretion, downregulation of Smad-mediated transcriptional activity and specificity protein 1 (*Sp1*) expression in tumor epithelial cells, suppression of downstream anti-apoptotic pathways (nuclear factor kappa-light-chain-enhancer of activated B cells (NF-*κ*B) and heat shock protein 70 (HSP70)), and ultimately a marked reduction in tumor growth and metastasis 105. Notably, selective suppression of actinin alpha 1 in CAFs by oroxylin A inhibits their contractility and matrix remodeling capacity, leading to a remarkable decrease of breast cancer lung metastasis owing to blockade of FAK/Src/JAK2/STAT3 signaling, reduced CCL2 secretion, and subsequent disruption of CAF-tumor cell crosstalk and metastatic niche formation 106. Critically, CAFs modulation may overcome therapy resistance, as demonstrated by improved chemotherapy efficacy through remodeling of the TME and FAP-dependent death receptor 5 hyper-clustering, which collectively restore apoptosis sensitivity 107, 108. These findings provide a crucial mechanistic rationale for targeting CAFs as a central regulatory target in cancer therapy.

## 3. Various nanomaterials for targeting CAFs

As mentioned above, CAFs form a dense stromal network that functions as both a physical and biochemical barrier, markedly restricting the penetration of conventional chemotherapeutics 109, 110. This CAF-centric obstacle is compounded by other biological hurdles, including the blood-brain barrier, the tumor vascular endothelium, and the complex stromal cell components (e.g., mesenchymal and immune cells), which collectively hinder deep-tissue drug penetration 111-114. Besides, conventional therapies using small-molecule drugs frequently suffer from poor stability, limited solubility, and off-target cytotoxicity, further hindering the therapeutic efficacy against tumor growth and metastasis 115, 116.

**Figure 2:**
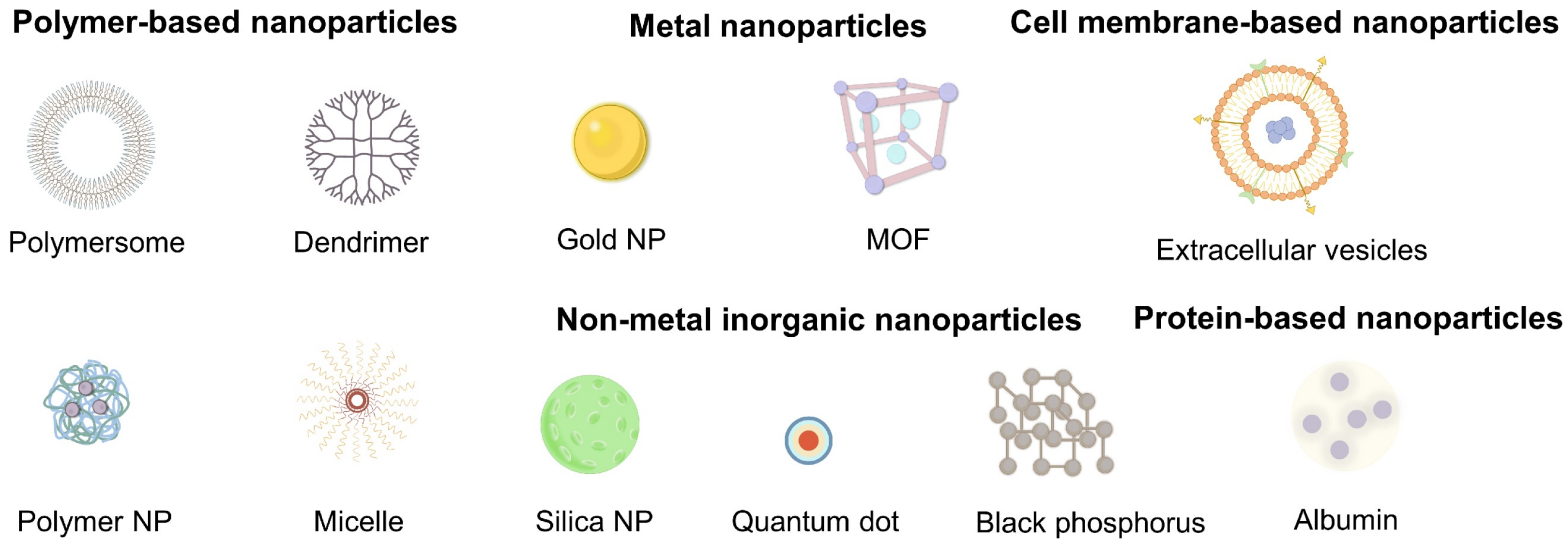
Various nanomaterials for targeting CAFs, including polymer-based, metal, cell membrane-based, non-metal inorganic and protein-based nanoparticles.

Nanomedicine has emerged as a rational counterstrategy. A variety of nanomaterials (e.g., polymers, metal and non-metal NPs, cell membrane-based NPs, protein-based NPs) with diverse compositions and functionalities have been developed for anti-cancer therapy (**Figure 2**) 117-120. Each kind of platform can be tailored for CAF-targeted delivery (**Table 1**). Polymer NPs provide exceptional design flexibility, allowing for the incorporation of multiple stimuli-responsive release mechanisms to achieve sophisticated multifunctional platforms. Their capacity to encapsulate and deliver a variety of payloads, including both hydrophobic and hydrophilic molecules, as well as cargoes with different molecular weights, such as small molecules, biomacromolecules, proteins, and vaccines, makes polymer NPs ideal for co-delivery applications 121. Meanwhile, metal NPs can also be endowed with specific cell targeting and multi-functionalization through surface modification with a variety of ligands 122, 123. Of specific note, owing to their unique optical as well as physicochemical properties, metal NPs can be employed for task-specific applications such as imaging and PTT 124, 125. In addition, cell membrane-based NPs offer innovative avenues for targeted drug delivery by mimicking the properties of natural cells, such as avoiding non-specific recognition and clearance by the immune system and prolonging blood circulation time 126. By precisely tuning size, charge, surface chemistry, and ligand density, NPs can 1) shield cargoes from degradation, 2) prolong circulation, and 3) achieve molecular recognition of CAF-expressed receptors, bypassing the unreliable EPR effect which is usually restricted by considerable inter-patient and intra-tumoral heterogeneity in vascular permeability, ECM density and interstitial fluid pressure 127-129. Quantitative meta-analyses demonstrate that the median tumor delivery of passively accumulating NPs is less than 1% of the injected dose and varies widely across patients and tumor types 62. The limitations of EPR-based targeting have accelerated the shift toward CAF-specific active targeting strategies on the basis of specific surface receptor recognition. Given that, the following section aims to provide a comprehensive and systematic overview of the various nanosystems designed for CAFs targeting, which categorizes nanomaterials not merely by composition but also by their surface functionalization strategies that are tailored to engage specific CAF markers, thereby bypassing the shortcomings of EPR-dependent delivery.

### 3.1 Polymer-based nanoparticles

Among various biomaterials employed for drug delivery, polymers have attracted a lot of attention due to the diversity of their composition as well as their multiple functionalities 130. A variety of structural designs for polymer-based NPs, such as liposomes, micelles, dendritic polymers, polymer-drug complexes or conjugates, have been explored for CAF-directed therapy 131, 132. Polymer NPs behave as programmable CAFs modulators whose tunable chemistry allows them to recognize CAF-restricted surface markers, neutralize CAF-secreted ECM proteins, and synchronize the release of CAFs modulators with cytotoxic agents. This integrated action redirects activated fibroblasts from tumor support to tumor restraint, converting the hostile stroma into a permissive environment for cancer therapy 133-135.

**Figure 3:**
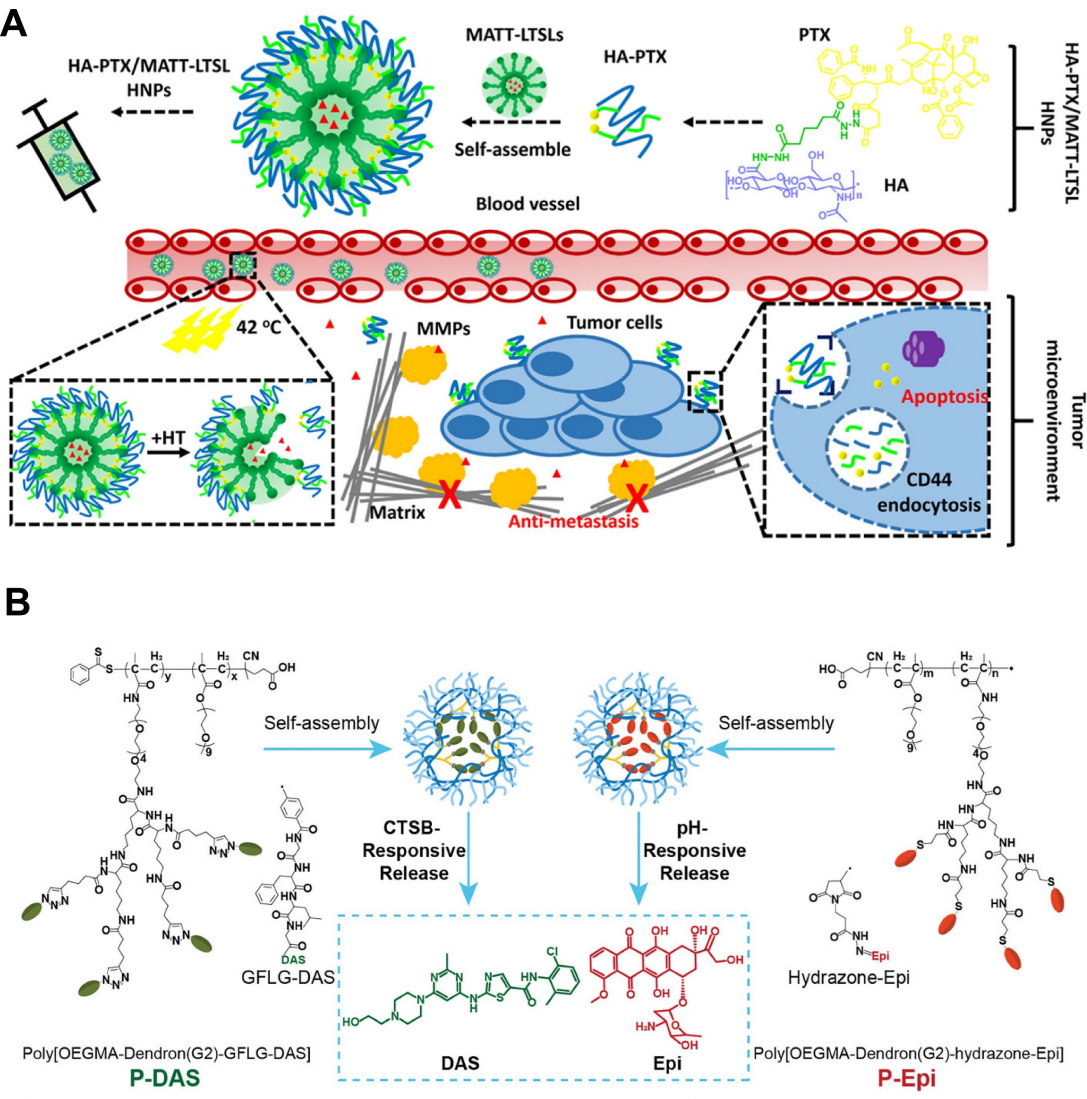
Polymer-based nanoparticles targeting CAFs. A) Schematic illustration of HA-PTX/MATT-LTSL HNP to dual target fibroblast activation and tumor cells for metastatic breast cancer treatment. Adapted with permission from 22, copyright 2018, American Chemical Society. B) Schematic of the preparation of P-DAS and P-Epi with tumor stimuli-responsive drug release to act on CAFs for ECM regulation and enhanced tumor treatment. Adapted with permission from 138, copyright 2024, Wiley-VCH.

For instance, Lv et al. developed a CAF-directed “lock-in and kill” polymer-liposome hybrid nanoplatform (HA-PTX/MATT-LTSL HNPs) that simultaneously suppressed CAFs activity and eradicated tumor cells (**Figure 3A**) 22. They loaded the broad-spectrum MMP inhibitor marimastat (MATT) into lysolipid thermosensitive liposomes (LTSLs), the surface of which was then coated with self-assembled hyaluronic acid-paclitaxel conjugates (HA-PTX). After intravenous injection, the resultant HNPs preferentially accumulated at tumor site via HA-mediated recognition, and the mild local hyperthermia of TME triggered rapid release of MATT from HNPs directly into CAF-rich stroma. MATT selectively inhibited CAF-secreted MMPs, resulting in a more than five-fold reduction in MMP-2/9 activity and a similar decrease in CAF-derived TGF-*β*1 expression, both of which were key drivers of ECM remodeling and fibroblast activation. Consequently, the stromal scaffold was preserved through restrained ECM degradation, effectively confining malignant cells within a CAF-restricted microenvironment. In orthotopic 4T1 tumors, this CAF-targeted strategy reduced *α*-SMA^+^ CAFs by 65% relative to controls. Unlike approaches that indiscriminately disrupt the TME to enhance NPs penetration that, in turn, may risk liberating metastatic cells, this CAF-directed “lock-in and kill” strategy boosted therapeutic efficiency by suppressing primary tumors and completely eradicating metastasis, offering an innovative route against highly metastatic breast cancer without compromising TME integrity.

Polymer-based NPs also have the potential to enhance the stability and circulation time of the nanodrugs for high tumor accumulation and retention 136. Among these, dendritic polymers have garnered interest due to their unique structures that enable multivalent interactions, which further extend CAF-centric applications by establishing sequential and spatially controlled delivery 137. As a typical example, Luo et al. developed dendritic polymers that sequentially delivered CAFs modulators and an immunogenic cell death (ICD) inducer to remodel the CAF-dominated stroma and enhance solid tumor therapy (**Figure 3B**) 138. Specifically, poly[OEGMA-Dendron(G2)-GFLG-DAS] (P-DAS) was synthesized by conjugating dasatinib (DAS) to dendritic poly(oligo(ethylene glycol) methyl ether methacrylate) (POEGMA) via a cathepsin B-cleavable Gly-Phe-Leu-Gly (GFLG) linker. Owing to the EPR effect and the small size (nearly 71 nm) of the resulting NPs, P-DAS preferentially extravasated and accumulated within the CAF-rich tumor stroma. Once internalized by CAFs, the GFLG linker was cleaved by cathepsin B that was highly expressed in these activated fibroblasts, liberating DAS to down-regulate *α*-SMA and collagen I synthesis and simultaneously suppress glycolytic flux by reducing the activity and expression of hexokinase and phosphofructokinase-1. This metabolic intervention reduced ECM density and relieved CAF-mediated immunosuppression through decreased lactate and arginine consumption. Additionally, an acid-responsive hydrazone bond conjugated Epi was tethered onto dendritic POEGMA to develop poly[OEGMA-Dendron(G2)-hydrazone-Epi], P-Epi, for ICD induction. After ECM remodeling by P-DAS, P-Epi penetrated tumors more efficiently. In orthotopic 4T1 breast cancer models, sequential administration of P-DAS followed by P-Epi reduced *α*-SMA-positive CAFs by 60% and collagen I deposition by 45%, enhanced cytotoxic T-lymphocyte infiltration, and achieved robust tumor regression without overt systemic toxicity. This sequential reprogramming of CAFs and subsequent immune activation acted synergistically to induce profound tumor regression and systemic anti-cancer immunity.

### 3.2 Metal nanoparticles

Metallic nanomaterials have emerged as a highly promising candidate for cancer therapy, becoming a focal point in both research and clinical applications 139, 140. Metal NPs, such as gold NPs and silver NPs exhibit a high specific surface area and excellent stability, enabling efficient drug loading via physical adsorption or chemical conjugation. This protects drugs from degradation and nonspecific adsorption during in vivo transportation, facilitating effective delivery 141. Additionally, some metallic NPs exhibit innate cytotoxicity, allowing them to serve directly as anti-cancer agents 142. Furthermore, through surface modifications, metallic nanomaterials have also been engineered to directly destroy the CAF-centric TEM. For instance, gold or silver NPs can be functionalized to selectively hitchhike onto CAFs and block signals such as TGF-*β*1 that normally fuel metastasis and immunosuppression. On the other hand, simultaneous release of chemotherapeutic payloads that are pre-adsorbed on the surface of metal scaffold can eradicate neighboring cancer cells 142-144. This CAF-targeting design elevates the NPs from a simple drug carrier to an active disruptor of the dialogue between CAFs and cancer cells, which simultaneously dismantles the pro-tumor stroma and enhances drug penetration.

**Figure 4:**
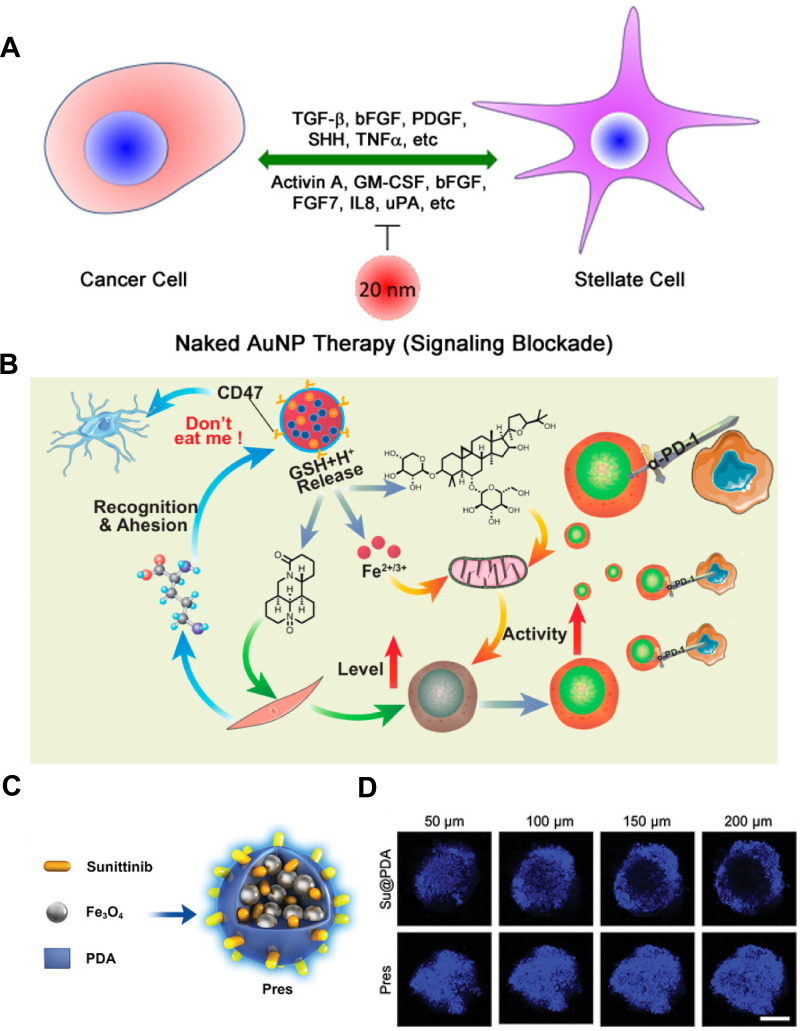
Metal nanomaterials targeting CAFs. A) Schematic of GNPs interference in the bidirectional communication between pancreatic cancer cells and pancreatic stellate cells. Adapted with permission from 143, copyright 2016, American Chemical Society. B) Schematic representation of the combination therapy of PmMN@Om&As against hepatocellular carcinoma (HCC) by inhibiting CAFs function in ECM production and increasing the level and activity of TILs simultaneously. Adapted with permission from 147, copyright 2023, American Chemical Society. C) Illustration of introducing Su and superparamagnetic Fe_3_O_4_ NPs into PDA for TME regression therapy through CAFs regulation. D) CLSM images of 4T1 and CAF-like 3T3 hybrid spheroids. (C-D) Adapted with permission from 148, copyright 2023, Wiley-VCH.

Gold NPs (GNPs) have been shown to induce the reprogramming of activated fibroblasts into a quiescent state, thereby remodeling the TME and inhibiting cancer growth 144. Hossen et al. demonstrated that GNPs could induce the expression of lipogenesis genes, such as fatty acid synthase (*FASN*), and sterol regulatory element-binding protein 2 (*SREBP2*), in CAFs 145. The endogenous synthesis of lipids in these cells helped maintain their quiescent phenotype, suggesting that GNPs could serve as an effective agent to modulate lipid metabolic pathways to functionally reshape the tumor stroma. A further study demonstrated that GNPs disrupted the bidirectional crosstalk between pancreatic cancer cells and pancreatic stellate cells (PSCs) by binding and sequestering key secreted proteins, such as TGF-*β* and FGF-2, thereby inhibiting their downstream signaling. This interference was further amplified through GNPs-induced endoplasmic reticulum stress, which activated the inositol-requiring enzyme 1 (IRE1)-dependent decay pathway and led to selective degradation of mRNAs encoding hub proteins. These actions collectively diminished PSC activation, repressed ECM gene transcription, and ultimately blocked matrix deposition within the tumor (**Figure 4A**) 143.

Harnessing a metal nanoplatform that actively targets CAFs, Kovács et al. designed a gold-core-silver-shell hybrid (Au@Ag) to probe how metal-based nanomaterials reshape the tumor-supportive activity of CAFs 142. In vitro, non-toxic doses of Au@Ag selectively applied to either NIH 3T3 cells or patient-derived CAFs abolished their ability to accelerate wound closure and invasion of 4T1 or MCF-7 tumor cells. RNA sequencing (RNA-seq) of Au@Ag-treated CAFs showed broad down-regulation of metastasis-related secreted factors, most notably secreted phosphoprotein 1 (Spp1) and pleiotrophin (Ptn). In orthotopic 4T1 mice, local peri-tumor injections of Au@Ag alone or combined with systemic doxorubicin (DOX) reduced intratumoral Spp1 protein and Ki67^+^ proliferating cancer cells within fibroblast-rich niches. Consequently, lung metastatic burden dropped without systemic toxicity, underscoring the value of the CAF-directed metal nanoplatform as a potent, clinically compatible adjunct to conventional chemotherapy.

Metal-organic frameworks (MOFs), with their porous structure, good biocompatibility and excellent chemical stability, hold great promise for biomedical applications. Their adaptable internal environment enables the loading of CAF-targeted payloads, making them attractive for controlled drug delivery in cancer therapy 146. As an example, Guo et al. constructed a multifunctional MOFs platform using magnetic Fe_3_O_4_ as a metal center capable of inhibiting CAFs function and turning “cold” tumors into “hot” tumors for enhanced immunotherapy (**Figure 4B**) 147. Leveraging the large surface area and pore volume, Fe_3_O_4_@MIL-100 MOFs was used to co-load two drugs with different polarities (i.e., oxymatrine (Om) and astragaloside IV (As)), followed by coating the surface with platelet membrane (Pm), which could not only evade mononuclear-phagocyte clearance but also bound avidly to CAF-secreted collagen. To this end, the resultant PmMN@Om&As could accumulate within the CAF-rich stroma, where Om inhibited CAFs activation while As restored mitochondrial fitness in exhausted tumor-infiltrating T lymphocytes (TILs). In orthotopic hepatocellular carcinoma-bearing mice, a single intravenous dose of PmMN@Om&As cut CAF-secreted collagen I by nearly 50% and tripled intratumoral CD8^+^ TIL density, driving 84% tumor regression when combined with anti-programmed cell death protein 1 (anti-PD-1). Likewise, Huang et al. exploited superparamagnetic Fe_3_O_4_ NPs to achieve magnetic targeting of the TME for CAFs regulation as well (**Figure 4C-D**) 148. As mentioned above, elimination of CAFs will loosen the rigid structure of the tumor, promote drug and lymphocyte infiltration, and return the immunosuppressive environment to the status before tumorigenesis coevolution. To fulfill such regression process, they simply co-assembled sunitinib (Su), superparamagnetic Fe_3_O_4_ NPs and polydopamine (PDA) into a 102 nm “Prune-to-Essence” nanoplatform (Pres) for TME regression therapy. Under the guidance of magnetic field, Pres was concentrated in the tumor region, where the delivered Su pruned neovessels and dampened CAFs survival signals by blocking VEGF/VEGFR2 and PDGFR-*β* pathways, thereby suppressing angiogenesis and CAF-driven ECM stiffening. In addition, PDA-mediated photothermal heating selectively eradicated *α*-SMA^+^ CAFs. This two-step nanostrategy significantly reshaped the TME in a multifaceted manner, which depleted 70% of *α*-SMA^+^ CAFs and softened the ECM, enabling 5-fold deeper drug penetration and eliciting systemic anti-cancer immunity. Such nanoplatforms underscore how metallic nanocarriers can simultaneously orchestrate CAFs reprogramming and immune infiltration, thus providing a desired therapeutic outcome for the treatment of solid tumors.

### 3.3 Non-metal inorganic nanoparticles: Silica, black phosphorus and carbon dots

**Figure 5:**
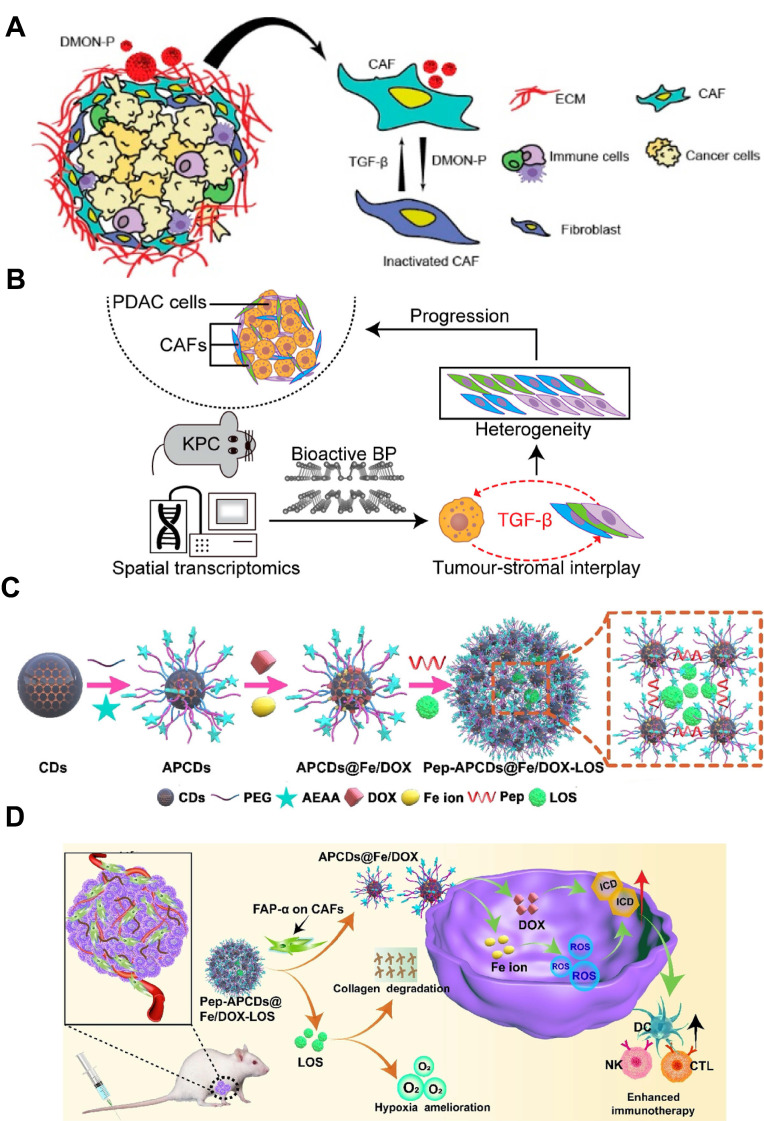
Non-metallic inorganic nanomaterials targeting CAFs. A) Reprogramming of CAFs by DMON-P to enhance the therapeutic effect of chemotherapy. Adapted with permission from 151, copyright 2024, American Chemical Society. B) Schematic representation of the bulk RNA sequencing for evaluating the effect of bioactive BP on CAFs and tumor-stromal interactions. Adapted with permission from 154, copyright 2024, American Chemical Society. C) Schematic illustration for the preparation of multiple drugs-loaded CD-based delivery nanosystem. D) The transformation and enhanced antitumor immunity mechanism of CD-based multistage delivery nanosystem. (C-D) Adapted with permission from 23, copyright 2024 Wiley-VCH.

Among various kinds of inorganic NPs, silica NPs have received much attention owing to their unique advantages, including easy tunability of particle size, porosity and structure, rendering them as suitable candidates for targeted drug delivery toward CAFs 149, 150. As an example, Yu et al. exploited this feature by embedding pioglitazone (Pio) inside dendritic mesoporous organosilica NPs (DMON-P) that carried tetrasulfide bridges (**Figure 5A**) 151. Once endocytosed by CAFs, the elevated intracellular glutathione (GSH) cleaved the bridges and liberated Pio directly in the cytosol. Released Pio activated PPAR-*γ*, antagonized TGF-*β*/Smad signaling and reprogrammed CAFs into a quiescent phenotype, markedly lowering *α*-SMA, collagen I and vimentin secretion. In subcutaneous 4T1 tumors, this CAFs normalization softened the ECM, leading to 2.8-fold enhancement of DOX penetration and 76% tumor regression, illustrating the engineering of silica nanomaterials to sense and dismantle the CAF-induced barrier for improved cancer therapy.

Black phosphorus (BP), an elemental phosphorus-based nanomaterial, has emerged as a promising bioactive platform for biomedical applications. Its cellular uptake and subsequent degradation can induce substantial changes in intracellular phosphate ion levels, which may play a crucial role in disease regulation 152, 153. Since signaling pathways involved in CAFs regulation are closely related to protein phosphorylation, Qu et al. revealed that bioactive BP could act as phosphate-ion “metabolic editors” within CAFs to remodel TME (**Figure 5B**) 154. Spatial transcriptomics and bulk RNA-seq revealed that BP down-regulated TGF-*β*1 expression in cancer cells by altering RNA splicing, and the reduced TGF-*β*1 secretion disrupted the TGF-*β*/Smad axis in neighboring CAFs. On the basis of this, BP treatment decreased both iCAFs and myCAFs subpopulations by nearly 60 %, suppressed *α*-SMA and collagen I/II deposition, and softened the ECM. By rebalancing CAFs heterogeneity and ECM composition, BP treatment converted the rigid, therapy-resistant stroma into a permissive environment, which was conducive to both drug penetration and immune cell infiltration.

Carbon dots (CDs), a type of carbon-based materials, exhibit excellent properties like high drug loading and good biocompatibility. Their sub-20 nm size facilitates penetration into the deep-seated regions of solid tumors 155, 156. Hou et al. exploited this feature by constructing a CAF-responsive honeycomb-like nanoassembly that sequentially delivered DOX, Fe ions and losartan (LOS) at distinct tumor sites (**Figure 5C-D**) 23. The platform was built by cross-linking aminoethyl anisamide (AEAA) (a targeting ligand of sigma receptor)-modified CDs with a FAP-*α*-cleavable peptide, followed by immobilizing DOX and Fe ions on its surface and encapsulating LOS within the mesopores. After systemic administration, the nanoplatform accumulated in stroma-rich tumors via the EPR effect and AEAA-mediated targeting, where the CAF-secreted FAP-*α* cleaved the responsive peptide for liberating LOS to suppress collagen synthesis and loosen the ECM. Concurrently, the residual DOX/Fe-loaded CDs with ultrasmall size of 10 nm penetrated deeply into the tumor and induced abundant reactive oxygen species (ROS) generation in tumor cells via Fenton reaction, causing severe ICD effect for eliciting anti-tumor immune response. As a result, such a CAF-targeting and multistage release strategy achieved 84.8% tumor inhibition and significant tumor metastasis suppression, demonstrating the effectiveness of CD-based nanocarriers in reprogramming CAF-dominated desmoplastic tumors for boosted tumor therapy.

### 3.4 Cell membrane-based nanoparticles

**Figure 6:**
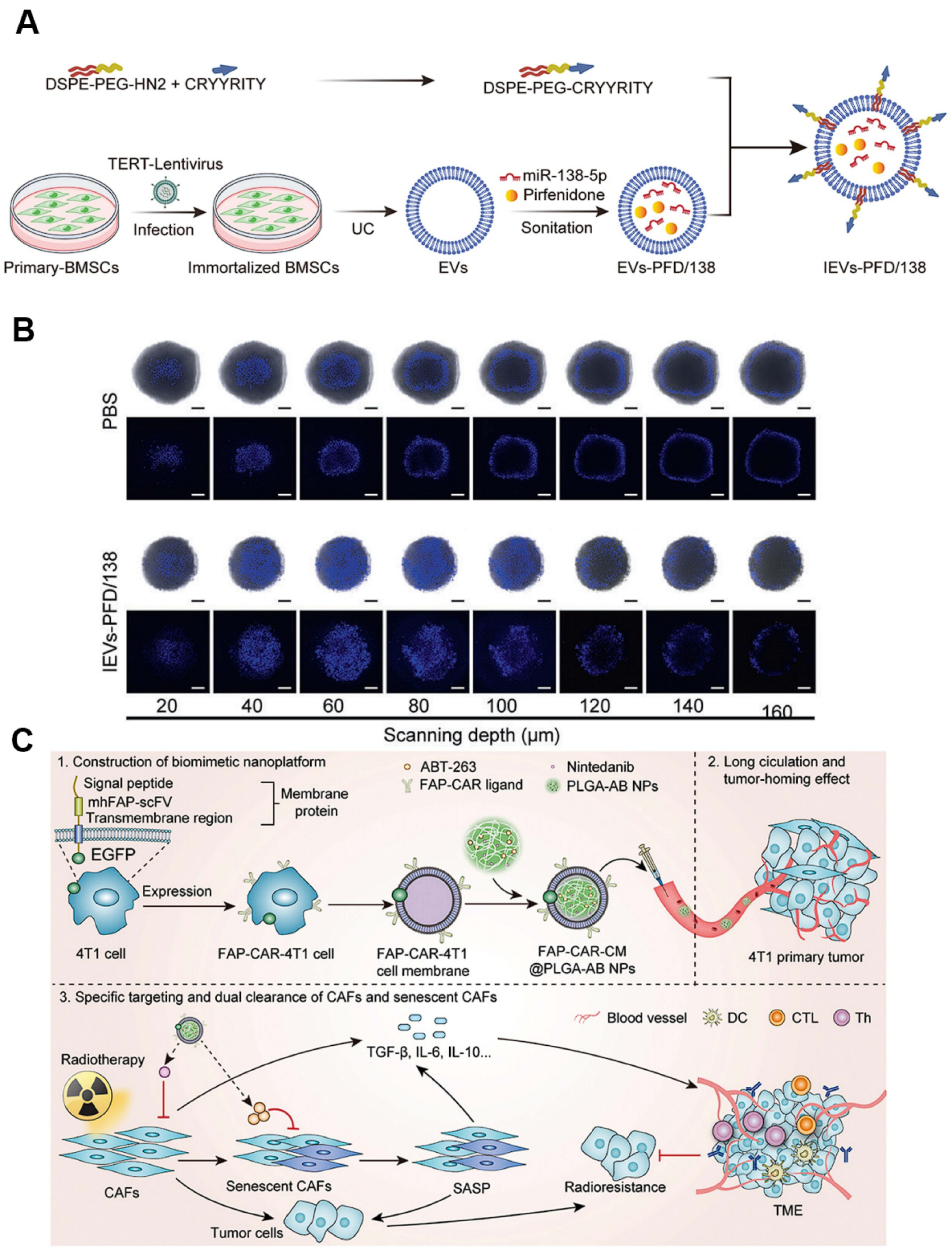
Cell membrane-based nanoparticles targeting CAFs. A) Schematic diagram of the BMSC-EV-based nanodevlivery system co-delivering antifibrotic drugs and miR-138-5p for targeted reprograming CAFs to enhance drug penetraion. B) IEVs-PFD/138 boosts Hoechst 33258 intensity and depth in stroma-rich 3D spheroids. (A-B) Adapted with permission from 163, copyright 2024, Nature Publishing Group. C) Schematic of the construction of tumor cell membrane-camouflaged nanocarrier for dual-targeted clearance of CAFs and senescent CAFs to remodel TME. Adapted with permission from 169, copyright 2024, Wiley-VCH.

EVs derived from living cells have emerged as promising cell membrane-based nanoplatforms for biomedical applications 157. These nanoscale vesicles characterized by a phospholipid bilayer enclosed structure, are capable of transporting bioactive molecules, such as proteins, nucleic acids, and lipids, as well as externally introduced small molecular drugs by loading the cargos in the inner cavity or on the lipid membranes with high efficiency to diseased sites 158, 159. Moreover, EVs usually exhibit favorable biocompatibility and low toxicity, rendering them as safe biomaterials for clinical translation. By virtue of EVs-based nanomedicine, the therapeutic index of conventional drugs can be greatly enhanced accompanying with minimized adverse effects 160, 161. For example, bone marrow mesenchymal stem cells (BMDCs) can differentiate into CAFs within the TME, and their autologous origin and homing affinity enable BMSC-derived EVs to preferentially reach and accumulate in CAF-rich stroma, thereby providing an ideal candidate for exploiting CAF-targeting delivery systems 162. On the basis of this, Zhou et al. loaded anti-fibrotic drugs pirfenidone (PFD) and miR-138-5p into BMSC-EVs and modified the NPs surface with integrin alpha5-targeting peptides to create a CAF-targeting and reprogramming nanomedicine (**Figure 6A**) 163. This nanoplatform specifically delivered the cargoes to CAFs, where miR-138-5p suppressed TGF-*β* signaling and proline-mediated collagen synthesis, cooperating with PFD to remodel the TME by reducing collagen production. This EV-based nanoplatform resulted in decreased tumor pressure and tripled gemcitabine penetration in deep-sited tumor regions (**Figure 6B**), which will be of great avail to boost the cell killing effect for effective cancer treatment.

Cell membrane-camouflaged NPs by coating synthetic NPs cores with natural cell membranes can endow the nanosystem with biomimic properties for use in various biomedical applications 164, 165. Notably, cell membrane-coated NPs can effectively retain the surface properties and functions of natural cell membranes, possessing outstanding features such as efficient immune evasion, prolonged circulation time, and enhanced homologous targeting activity. These attributes have been widely leveraged for CAF-targeted drug delivery 166, 167. As an example, Zang et al. utilized a hybrid membrane derived from cancer cells and activated fibroblasts to coat solid lipid NPs containing the glycolysis inhibitor PFK15 and the chemotherapeutic drug PTX, thereby constructing a biomimetic nanocarrier that targeted both cancer cells and CAFs simultaneously 168. Specifically, PFK15 delivered in CAFs inhibited their metabolic activity for producing lactate, thereby abolishing the lactate-supported tumor growth, while paclitaxel exerted direct cytotoxicity in tumor cells. This dual metabolic and cytotoxic intervention enhanced chemosensitivity and achieved 80% tumor growth inhibition in desmoplastic breast tumors. Similarly, Chen et al. incorporated FAP-binding receptor into a biomimetic core-shell nanoplatform based on cell membrane-coating nanotechnology for dual-targeted elimination of CAFs and senescent CAFs (sCAFs) (**Figure 6C**) 169. The resulting FAP-CAR-CM@PLGA-AB selectively eliminated both conventional and senescent CAFs, reduced TGF-*β* secretion and collagen deposition, and restored T cell activation. When combined with radiotherapy, the strategy achieved complete tumor regression in 50% of mice and overcame CAF-driven radio-resistance without detectable systemic toxicity.

### 3.5 Protein-based nanoparticles

**Figure 7:**
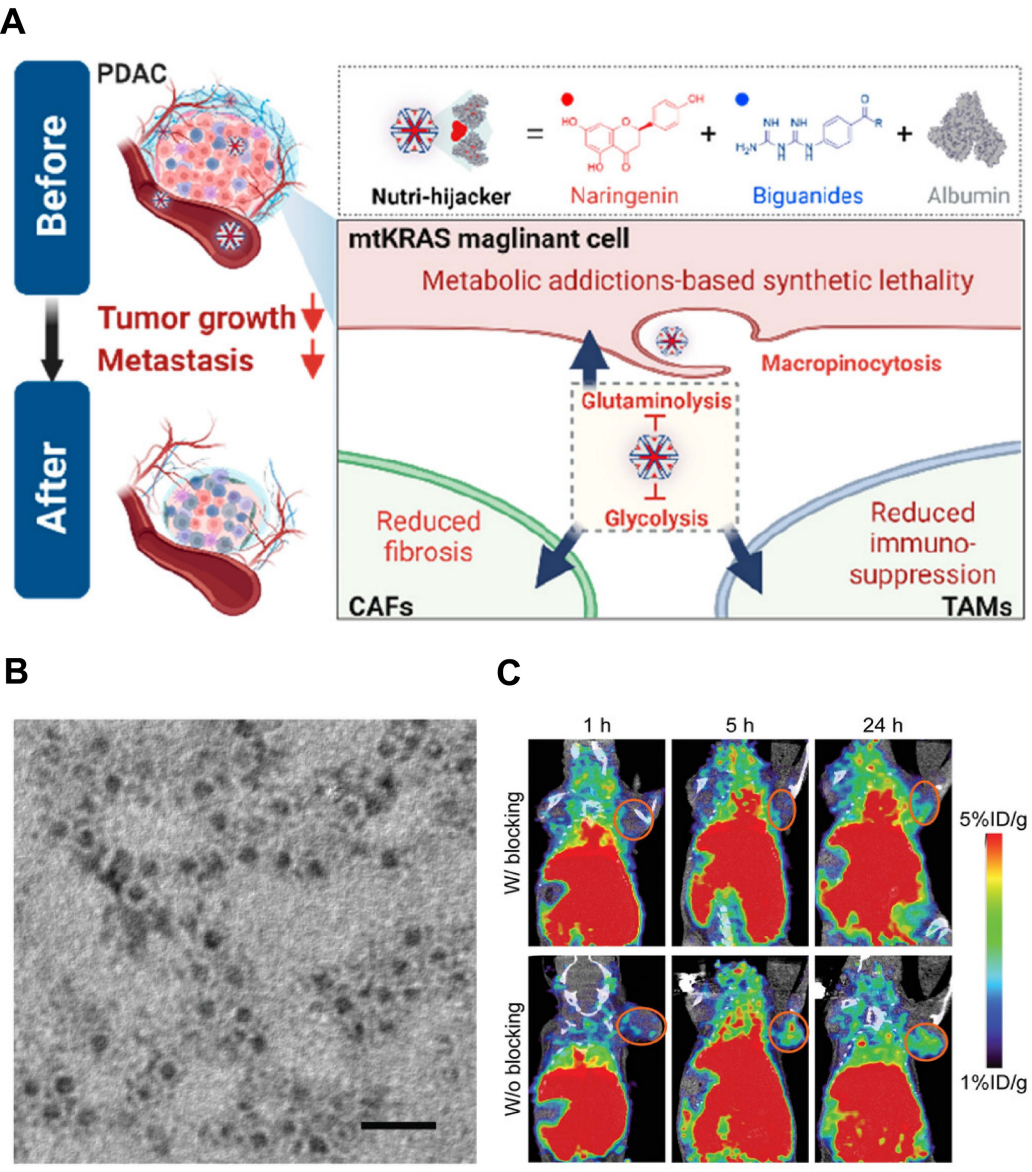
Protein-based nanoparticles targeting CAFs. A) The construction of “Nutri-hijacker” based on albumin for metabolic addiction-based synthetic lethality by acting on CAFs, TAMs, and magniligant cells to effectively suppresses the growth of mtKRAS cancer cells, reduces tumor fibrosis, and alleviates immunosuppressive conditions. Adapted with permission from 173, copyright 2023, American Chemical Society. B) TEM image and C) the in vivo tumor targeting ability of the ferritin-based nanoplatform of *α*FAP-Z@FRTs for permitting site-specific eradication of CAFs. (B-C) Adapted with permission from 176, copyright 2020, Wiley-VCH.

Nanoscale proteins with specific three-dimensional structure can be easily combined with other kinds of proteins, genetic materials and small molecules to form function-enhanced nanocomplexes. These protein-based NPs can be re-configured as CAFs intercepting shuttles that deliver therapeutic agents to activated fibroblasts 170, 171. For example, albumin, a major source of nutrients, is a commonly used protein-carrier that can interact with the secreted protein acidic and rich in cysteine (SPARC) in tumor tissue to mediate the active targeting for facilitated tumor accumulation 172. In a therapeutic strategy targeting malignant cells with Kirsten rat sarcoma viral oncogene homolog mutations (mtKRAS), Chen et al. exploited albumin's intrinsic affinity for the SPARC-rich ECM to construct the “Nutri-hijacker”, an 82 nm albumin nanocomplex that co-loaded the glycolysis inhibitor biguanide and the glutaminolysis blocker naringenin (**Figure 7A**) 173. After intravenous injection, the nanocomplex accumulated in the tumor via SPARC-mediated anchorage and was then internalized by both KRAS-mutant cancer cells and CAFs through macropinocytosis. In CAFs, naringenin blocked TGF-*β*/Smad2/3 signaling, resulting in the reduction of fibronectin, *α*-SMA and collagen secretion by 59%, 79% and 76%, respectively. This metabolic reprogramming repressed CAFs activation and softened the stroma, while the concurrent nutrient starvation of cancer cells further amplified the anti-fibrotic effect. As a result, the Nutri-hijacker remarkably remodeled CAF-mediated TME for improved drug penetration and alleviated immunosuppression, achieving marked suppression of tumor growth and metastasis in orthotopic mouse models.

Ferritin NPs, assembled from 24 subunits into a unique cage-like structure, have an overall dimension of about 12 nm and an internal cavity diameter of approximately 8 nm. This structure endows ferritin NPs with considerable potential for CAF-targeting therapy 174, 175. For example, Xie et al. developed using FAP-specific single-chain fragment variable (scFv)-conjugated apoferritin NPs with the loading of photosensitizer ZnF16Pc (denoted as *α*FAP-Z@FRT) for targeted PDT (**Figure 7B-C**) 176. After intravenous injection, *α*FAP-Z@FRT selectively bound to FAP^+^ CAFs within tumors and eradicated them by cytotoxic ¹O_2_ generated under 671 nm laser irradiation, leading to reduced collagen deposition by 76% for disrupting the ECM barrier. Moreover, the CAFs depletion also triggered CD8^+^ T cell-mediated anti-CAFs immunity that suppressed both primary and distant tumors, which significantly prolonged the overall survival by further synergizing with anti-PD-1 therapy. Such an *α*FAP-Z@FRT PDT approach provides an effective strategy for permitting site-specific CAFs elimination to retard the growth of malignant tumors.

## 4. Nanostrategies targeting CAFs in cancer therapy

**Figure 8:**
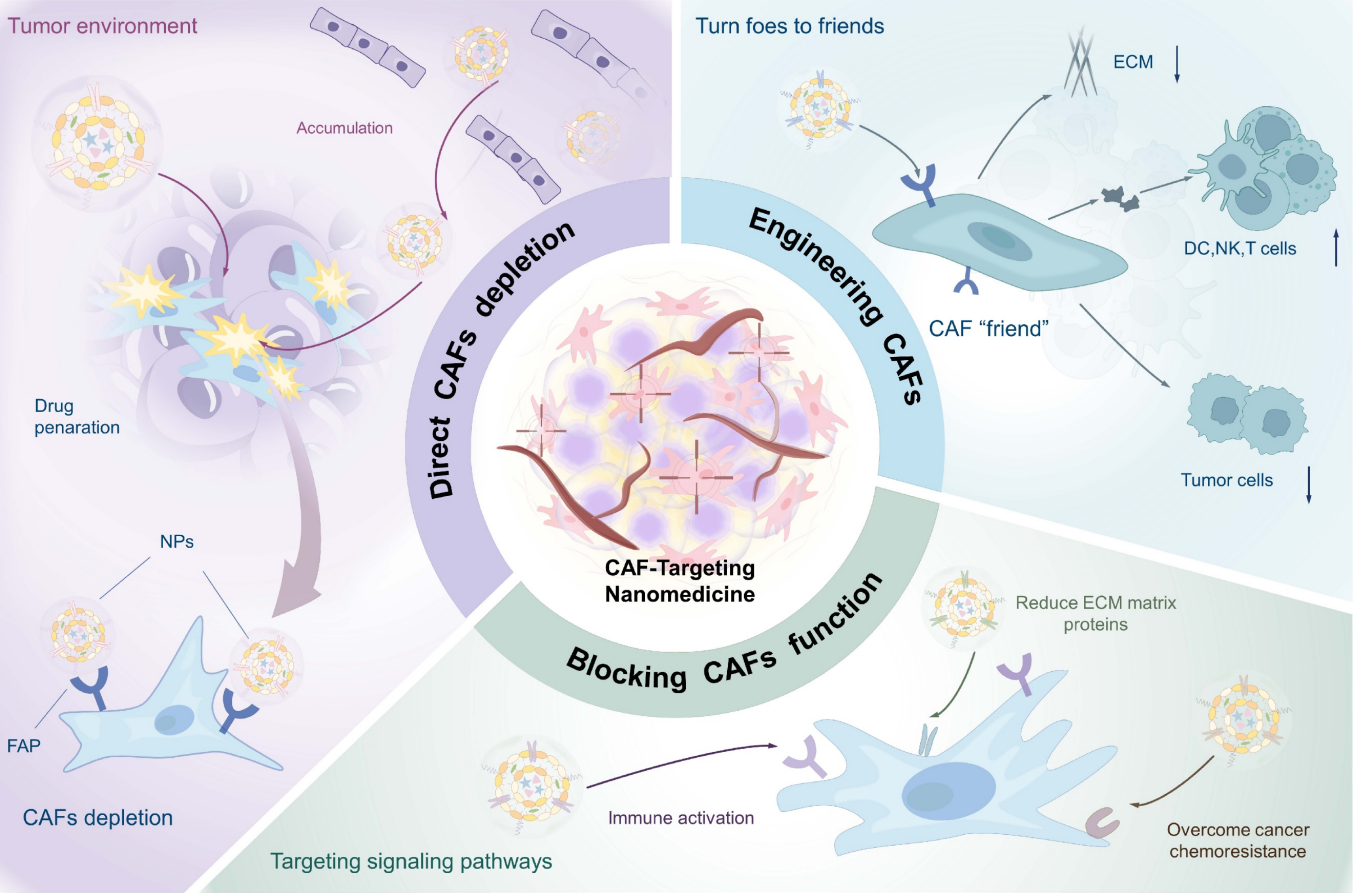
CAF-targeted nanomedical strategies, including direct depletion of CAFs, engineering CAFs to turn foes into friends and blocking CAFs' functions, aiming to remodel ECM of stromal tumors, improve drug penetration, enhance immune response, and overcome chemoresistance in cancer treatment.

As predominant stromal cells in TME, activated CAFs play a crucial role in inducing tumor fibrosis, which significantly impedes the penetration and efficacy of nanomedicines. Furthermore, the interaction between CAFs and tumor cells not only facilitates tumor growth and metastasis but also contributes to the establishment of an immunosuppressive environment 177, 178. With growing insights into the biology of CAFs, diverse therapeutic strategies targeting CAFs have emerged. These include the selective elimination of specific CAFs subpopulations, functional modulation of CAFs to convert them into tumor-suppressive phenotype, and cellular reprogramming to convert “bad” CAFs into therapeutic “friends” **(Table 2)**. Accordingly, this section provides a structured discussion of nanomedical CAFs targeting strategies, presenting: 1) direct CAFs elimination, 2) targeting specific signaling pathways in CAFs to reverse their function, and 3) engineering CAFs, with emphasis on seminal studies** (Figure 8)**.

### 4.1 Direct CAF elimination

CAFs constitute the largest stromal cell population and are major producers of ECM components in the TME. Their rapid proliferation in response to tumor growth generates solid stress that impedes the penetration of nanotherapeutics, thereby limiting treatment efficacy 179. Under such circumstances, anti-stromal strategies aimed at directly eliminating ECM-producing CAFs show considerable potential for overcoming stromal barriers and enhancing drug perfusion.

**Figure 9:**
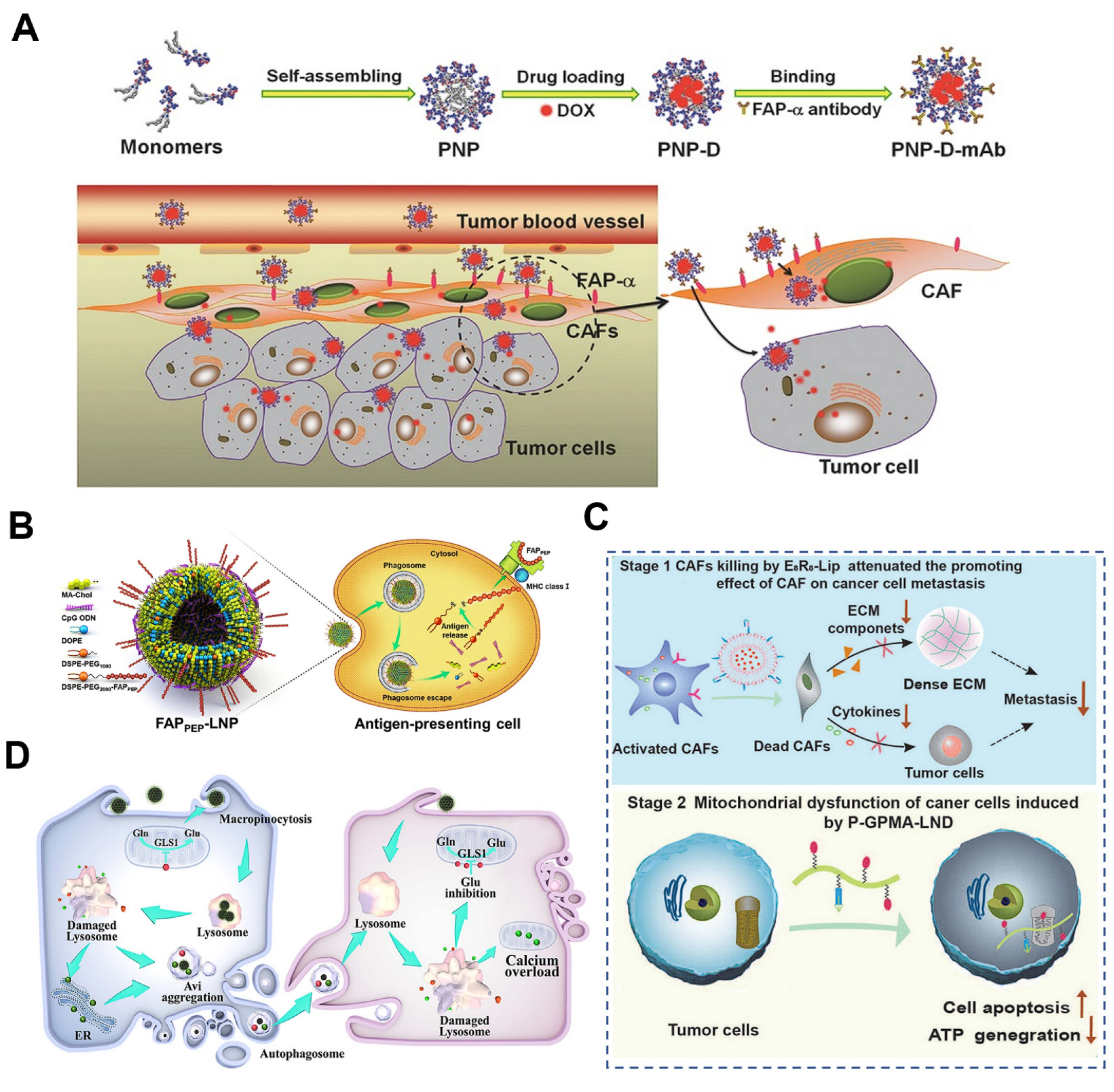
Direct CAFs elimination for overcoming stromal barriers. A) Construction of DOX-containing self-assembled peptide NPs modified with mAb (PNP-D-mAb) for targeted elimination of CAFs to promote drug penetration. Adapted with permission from 59, copyright 2015, Wiley-VCH. B) Schematic illustration of the design of the FAP_PEP_-SLNP nanovaccine and its antitumor efficacy. Adapted with permission from 56, copyright 2023 American Chemical Society. C) Schematic illustration of the combination of CAF-depleting liposome (E_8_R_8_-Lip) and mitochondria-damaging polymer (P-GPMA-LND) for inhibiting tumor metastasis. Adapted with permission from 186, copyright 2021 Wiley-VCH D) Schematic illustration of DOX@CG-CaPDA nanoplatform to enhance drug penetration by triggering CAFs to release drug-encapsulated autophagosomes for subsequent internalization by tumor cells. Adapted with permission from 24, copyright 2025, American Chemical Society.

As previously noted, CAF-specific surface biomarkers represent promising targets for therapeutic intervention 180. Engineering targeting aptamers and antibodies can improve the precision of CAFs depletion 181. For example, Ji et al. developed a core-shell structured NP (PNP-D-mAb) with the loading of DOX and conjugating a mAb against FAP-*α* (**Figure 9A**) 59. Given the selective expression of FAP-*α* on CAFs membranes, PNP-D-mAb specifically bounded to CAFs and released DOX intracellularly, resulting in direct CAFs depletion. In 4T1 tumor-bearing BALB/c mice, intravenous administration of PNP-D-mAb reduced *α*-SMA^+^ CAFs by 62% and collagen I by 45%, while doubling DOX penetration depth in solid tumor, offering a promising strategy for treating CAF-rich solid tumors. Complementing antibody-guided elimination, Shin et al. developed a pan-tumor nanovaccine that could eradicate FAP-expressing CAFs via adaptive immunity (**Figure 9B**) 56. They predicted and selected two immunodominant FAP-specific epitope peptides in the computer, which were displayed on the surface of lipid NPs that were encapsulated with CpG adjuvant to form the nanovaccine (FAP_PEP_-SLNPs). Immunization with FAP_PEP_-SLNPs not only depleted FAP^+^ CAFs but also elicited robust peptide-specific CD8^+^ T-cell responses. Consequently, intratumoral accumulation of co-administered chemotherapeutics increased, producing marked regression across multiple tumor models.

Given the role of CAFs in promoting hypoxia and metastasis, a sequential dual-targeting strategy has been proposed to address these challenges. Hypoxia drives cancer cell intravasation and premetastatic niche formation, facilitating metastasis 182. CAFs, as major stromal components, contribute significantly to hypoxia via ECM production and through robust mitochondrial aerobic respiration in tumor cells 183. Additionally, CAFs secrete various factors such as IL-6 and VEGF that promote dissemination, while mitochondrial aerobic respiration in cancer cells provides energy for tumor proliferation and metastasis as well 184, 185. To this end, interfering with CAFs and mitochondria simultaneously exhibits unique advantages to reverse hypoxic tumor and suppress metastasis. Thus, Yi et al. introduced a sequential, dual-targeted nano-strategy that first depleted CAFs and then disrupted cancer cell mitochondria to break both “soil” and “seed” components of metastasis (**Figure 9C**) 186. They engineered a CAF-responsive liposome (E_8_R_8_-Lip) carrying DOX and FAP-*α*-cleavable E_8_R_8_ peptide. Upon exposure to CAF-secreted proteases, the shielding anionic E_8_ domain was removed, unmasking the cationic R_8_ cell-penetrating peptide and driving selective uptake into CAFs, resulting in a subsequent 70% reduction in intratumoral *α*-SMA^+^ CAFs, a marked decrease in ECM deposition, and a consequent rise in tumor oxygenation. On the other hand, lonidamine (LND), a potent aerobic respiration inhibitor, was conjugated onto the water-soluble N-(2-hydroxypropyl) methacrylamide (HPMA) copolymers with the side chains modified with the guanidine groups to generate a mitochondria-targeted polymer of P-GPMA-LND, which significantly reduced the energy supply and increased apoptosis in cancer cells. Orthotopic 4T1 mice treated sequentially with E_8_R_8_-Lip and P-GPMA-LND (i.v.) showed 70% fewer CAFs and a 5-fold drop in lung metastases. As such, the two-pronged approach of CAFs depletion and mitochondria dysfunction led to excellent anti-metastatic effect.

Certain physical intervention modalities have demonstrated efficacy in tumor combination therapy and exhibit considerable potential for CAFs treatment as well. For instance, Feng et al. showed that Ca^2+^-doped polydopamine nanozymes (DOX@CG-CaPDA) could induce rapid temperature increase to 50 °C under 808 nm light irradiation for effective PTT, which could cause direct lysis of CAFs and disintegration of the collagen meshwork (**Figure 9D**) 24. The dying CAFs released drug-encapsulated autophagosomes, which were then internalized via macropinocytosis by adjacent glutamine-starved cancer cells, thereby enhancing drug penetration throughout 4T1 tumors.

Beyond their therapeutic potential, CAFs have already entered the clinic as an imaging target. ^68^Ga-labelled FAP inhibitors (FAPI) are now standard positron emission tomography-computed tomography (PET-CT) tracers, leveraging the near-universal FAP overexpression on CAFs to achieve high tumor-to-background contrast and sensitive detection of primary and metastatic lesions across multiple cancer types 187. Building on this translational momentum, CAF-directed nanoprobes are emerging as next-generation imaging tools. Wen et al. offered a more comprehensive demonstration of how nanoscale engineering could repackage the FAPI paradigm into a fully theragnostic platform 25. They synthesized ultrasmall PEGylated melanin NPs, the surface of which was functionalized with FAPI for CAFs targeting and the core of which was simultaneously incorporated ^64^Cu (for PET), Mn^2+^ (for high-relativity magnetic resonance imaging), and ^131^I (for *β*-emitting radionuclide therapy). In a CAF-rich glioblastoma model, the resultant NPs demonstrated rapid, FAP-dependent accumulation and prolonged intratumorally retention. More importantly, the multiplexed nanosystem enabled synergistic targeted CAFs depletion and PTT under 808 nm irradiation, yielding 79% tumor growth inhibition. By integrating CAF-specific targeting, multimodal imaging, and internal *β*-radiation into a single nanocarrier, this work achieved improved pharmacokinetics, tunable isotope payloads and real-time monitoring of stromal depletion, which exemplified the distinct advantages that nanomedicine for advanced FAPI-based theragnostic.

Building on the same radiobiological rationale, external beam radiotherapy can be synergistically boosted by CAFs depletion. Gong et al. developed a microbubble-inspired oxygen-transporting multi-fluorocarbon nanosystem loading DiIC18(5) (DiD) and halofuginone (M-FDH) for boosting radiotherapy 188. Perfluorocarbons were used as artificial oxygen carriers for alleviating hypoxia due to their high oxygen-carrying capacity and good biocompatibility. In the designed nanosystem, the radiosensitizer, DiD, which could effectively generate reactive ROS, was loaded for inducing radiation cell-killing effect, and stroma regulator halofuginone, which was aimed to destroy the stromal barrier, was loaded to relieve tumor hypoxia. Upon X-ray radiation, the perfluorocarbon-based nanoplatform caused efficient production of ROS followed by the induction of ICD. With the combination of CAFs elimination and ECM decrease for efficient oxygen supply, effective killing effect of cancer cells by radiation therapy and anti-tumor immune response was achieved. Thus, whether through internal radionuclide therapy or external beam sensitization, CAF-targeted nanomedicine provides a unified platform to overcome both physical and biological barriers, translating the unique biology of CAFs into versatile therapeutic leverage.

Despite the promise of CAFs depletion via surface markers, several limitations remain. Certain CAFs subpopulations may inhibit tumor invasion and metastasis 189, 190. Indiscriminate depletion could harm these beneficial subsets and disrupt tumor suppression. Moreover, CAFs interact extensively with other TME cells, such as immune cells and tumor cells. Their elimination may trigger unpredictable stromal reactions that affect other therapies. Therefore, future nanostrategies would be directed to selectively target pro-tumorigenic CAFs subpopulations while preserving tumor-restraining functions. An emerging alternative is to reprogram CAFs into a quiescent or tumor-suppressive state rather than indistinctively eliminating them, enabling more precise and effective therapeutic outcomes.

### 4.2 Targeting specific signaling pathways in CAFs to reverse their function

Given the challenges of direct CAFs depletion, targeting cytokines or key regulators driving CAFs biology offers a promising alternative. CAFs are activated by specific stimuli within the TME and exert pro-tumorigenic effects through multiple mechanisms, including ECM production, cytokine secretion, immunosuppression, and promotion of tumor cell proliferation and metastasis 191, 192. Modulating specific signaling pathways can reverse the pro-tumorigenic functions and enhance therapeutic efficacy 193.

**Figure 10:**
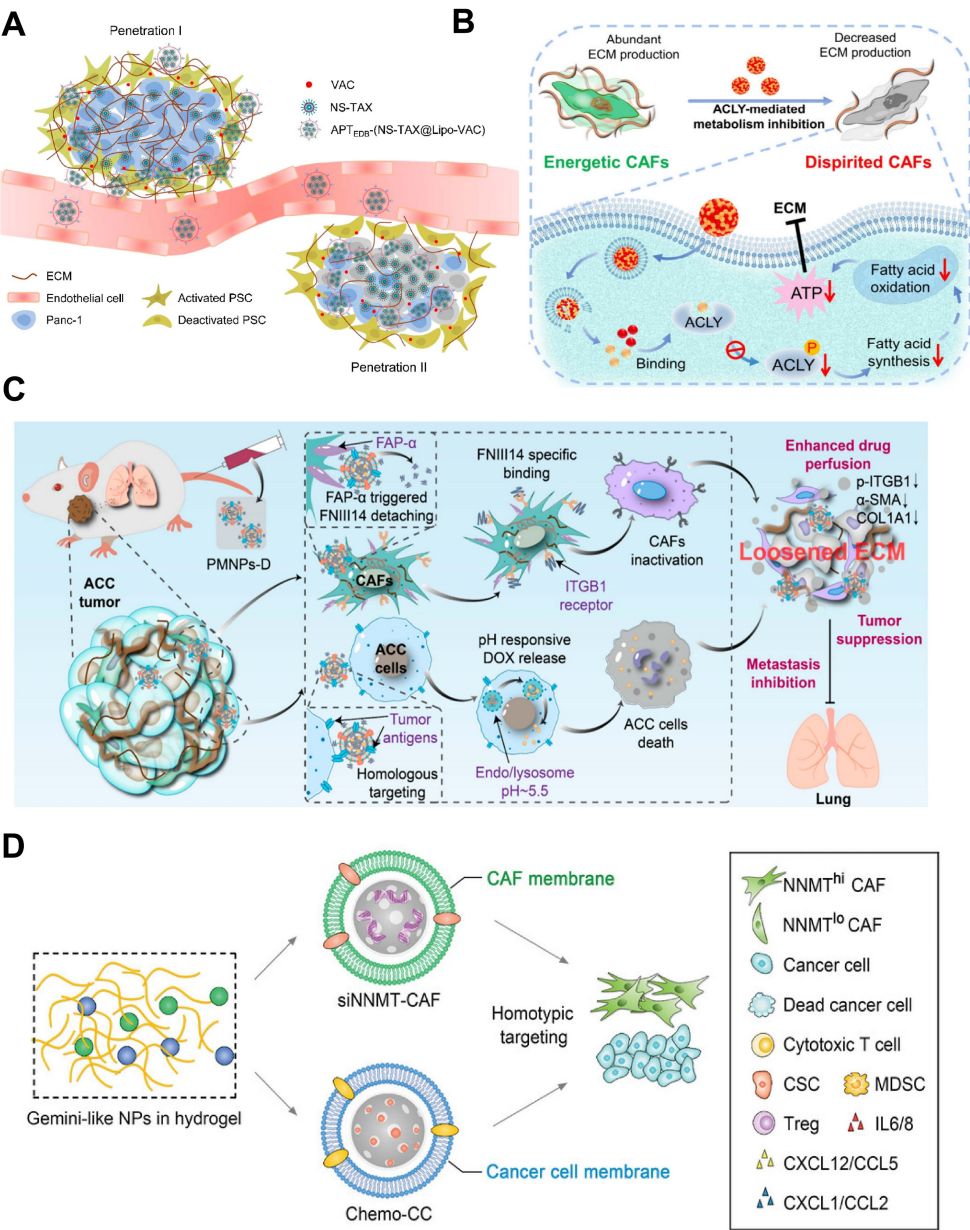
Targeting specific signaling pathways in CAFs for reversing their functions. A) Schematic of penetration cascade of the size switchable NS-TAX@Lipo-VAC in pancreatic desmoplastic stroma. Adapted with permission from 195, copyright 2021, American Chemical Society. B) Schematic of the construction of carrier-free nanoagent interfering with ACLY-mediated cellular metabolism in CAFs to promote tumor penetration. Adapted with permission from 196, copyright 2024, American Chemical Society. C) Schematic of the reversion of CAFs' function in ECM production to promote drug penetration in tumors by FNIII14 peptide-enriched membrane nanocarrier. Adapted with permission from 197, copyright 2023, American Chemical Society. D) Schematic of a hydrogel platform for co-delivery of CAF- and cancer cell-targeted NPs to modulate *NNMT*-assocated metabolism in CAFs for overcoming chemoresistance and immunosuppression. Adapted with permission from 201, copyright 2023, Wiley-VCH.

As major ECM producers, CAFs create a physical barrier that shelters tumor cells and impedes nanomedicine penetration 194. Accordingly, disrupting the matrix barrier is crucial for improving cancer treatment. Given that, Zhao et al. designed a size switchable nanosystem (NS-TAX@Lipo-VAC) to deliver vactosertib (VAC) (an inhibitor against TGF-*β* signaling) and chemotherapeutic drug paclitaxel (TAX) to overcome stromal barriers (**Figure 10A**) 195. In their study, the small nanospheres carrying TAX (NS-TAX) were encapsulated into the hydrophobic core of VAC-loaded liposomes (Lipo-VAC), and a peptide (APTEDB) targeting the fibronectin extra domain B (EDB) was used to be modified on the outer surface of NPs in order to anchor the NPs to abundant tumor-associated fibronectin in cancer stroma. In the desmoplastic pancreatic model, APTEDB-(NS-TAX@Lipo-VAC) (size nearly 200 nm) could targeted accumulate in the tumor region after blood circulation and deliver VAC in CAFs to specifically act on TGF-*β* signaling, leading to significantly reduced expression of ECM proteins in the tumor stroma after collapse of the liposomes. Concurrently, small-sized NS-TAX (nearly 40 nm) could be released and penetrated deeply into the tumor for effective tumor cell-killing. Such a cascade penetration strategy provides an effective anti-stromal approach for deep drug penetration to enhance the therapeutic effectiveness.

In our recent work, a carrier-free nanoagent (CFNA) by the self-assemble of a specific inhibitor, NDI-091143, with chemotherapeutic drug molecules (DOX or PTX) was designed to target on CAFs for deep drug penetration (**Figure 10B**) 196. The delivery of NDI-091143 could specifically inhibit the ATP-citrate lyase (ACLY)-mediated cellular metabolism in CAFs, resulting in the blocking of subsequent synthesis of fatty acids, which closely involved in the energy metabolism in cells. As a result, the generation of energy in CAFs was seriously suppressed, making these cells in a dispirited state that were unable to produce abundant ECM. On the basis of this, the drugs were deeply transported into the inner core of tumor, and the therapeutic effect of chemotherapeutic drugs was greatly improved. In another work, we found that integrin *β*1 (ITGB1) was significantly overexpressed on CAFs and was responsible for forming the dense tumor stroma, and a specific peptide (FNIII14) derived from fibronectin was demonstrated to act on ITGB1 to induce the inactivation of CAFs. We, therefore, constructed a FNIII14 peptide-enriched membrane nanocarrier (designated as PMNPs-D) to reverse the function of CAFs in ECM generation, thereby disrupting stromal barrier for deep drug penetration (**Figure 10C**) 197. By evaluating in an aggressive adenoid cystic carcinoma tumor-harboring mice model, PMNPs-D showed substantial tumor inhibition and metastasis retardation.

Besides, CAFs secrete immunosuppressive chemokines such as CCL2 and, together with cancer stem cells (CSCs), promote chemoresistance 198. Nevertheless, direct depletion of CAFs may inadvertently enhance tumor invasiveness and metastasis 199. A more refined approach targets the metabolic enzyme nicotinamide N-methyltransferase (*NNMT*), whose over-expression in CAFs and cancer cells depletes nicotinamide (vitamin B3) and the methyl donor S-adenosylmethionine (SAM), would achieve epigenome reshaping to sustain a pro-tumoral niche 200. As such, Guo et al. reported a two-pronged cancer cell elimination and CAFs transformation nanostrategy for tumor therapy (**Figure 10D**) 201. Specifically, the TME-responsive hydrogel was used to load two kinds of Gemini-like NPs constructed from mesoporous silica NP (MSN) cores: one was loaded with *NNMT* short interfering RNA and coated with CAF-derived membrane (si*NNMT*-CAFs); the other one was camouflaged with cancer cell membrane for the delivery of chemotherapeutic drugs (Chemo-CC). Upon homotypic targeting, si*NNMT*-CAF downregulated *NNMT* expression in CAFs, restored SAM and NAD^+^ levels, and epigenetically silenced the secretion of pro-stemness factors (IL-6, IL-8, CCL2) that normally sustain the CD44^+^CD133^+^ cancer stem cell (CSC) niche; concurrently, Chemo-CC eliminated tumor cells with high efficiency. As a result, the two Gemini-like NPs in hydrogel cut CAF-secreted CCL2 by 72%, reduced CD44^+^CD133^+^ CSCs by 68%, and tripled intratumoral granzyme-B^+^ CD8^+^ T cells, yielding almost complete tumor regression in 90% of mice (n = 10), which achieved a remarkable regression of the chemo-resistant tumors.

Targeting specific cytokines or regulatory factors would allow precise inhibition of pro-tumorigenic CAF functions, thereby minimizing collateral damage. Nevertheless, the high heterogeneity and plasticity of CAFs pose significant challenges. CAFs rely on diverse signaling pathways for activation, and targeting a single factor may not comprehensively suppress their functions. Moreover, certain key cytokines (e.g., TGF-*β* and IL-6) play roles in systemic immunity and physiology, and their inhibition may cause immune dysregulation or side effects 202, 203. Thus, reversing CAFs function requires a more nuanced approach that makes allowance for heterogeneity, systemic effects, and resistance mechanisms. Identifying specific CAFs biomarkers and signaling pathways would be more promising in further research for precise and effective therapies.

### 4.3 Engineering CAFs: turning enemies into friends

**Figure 11:**
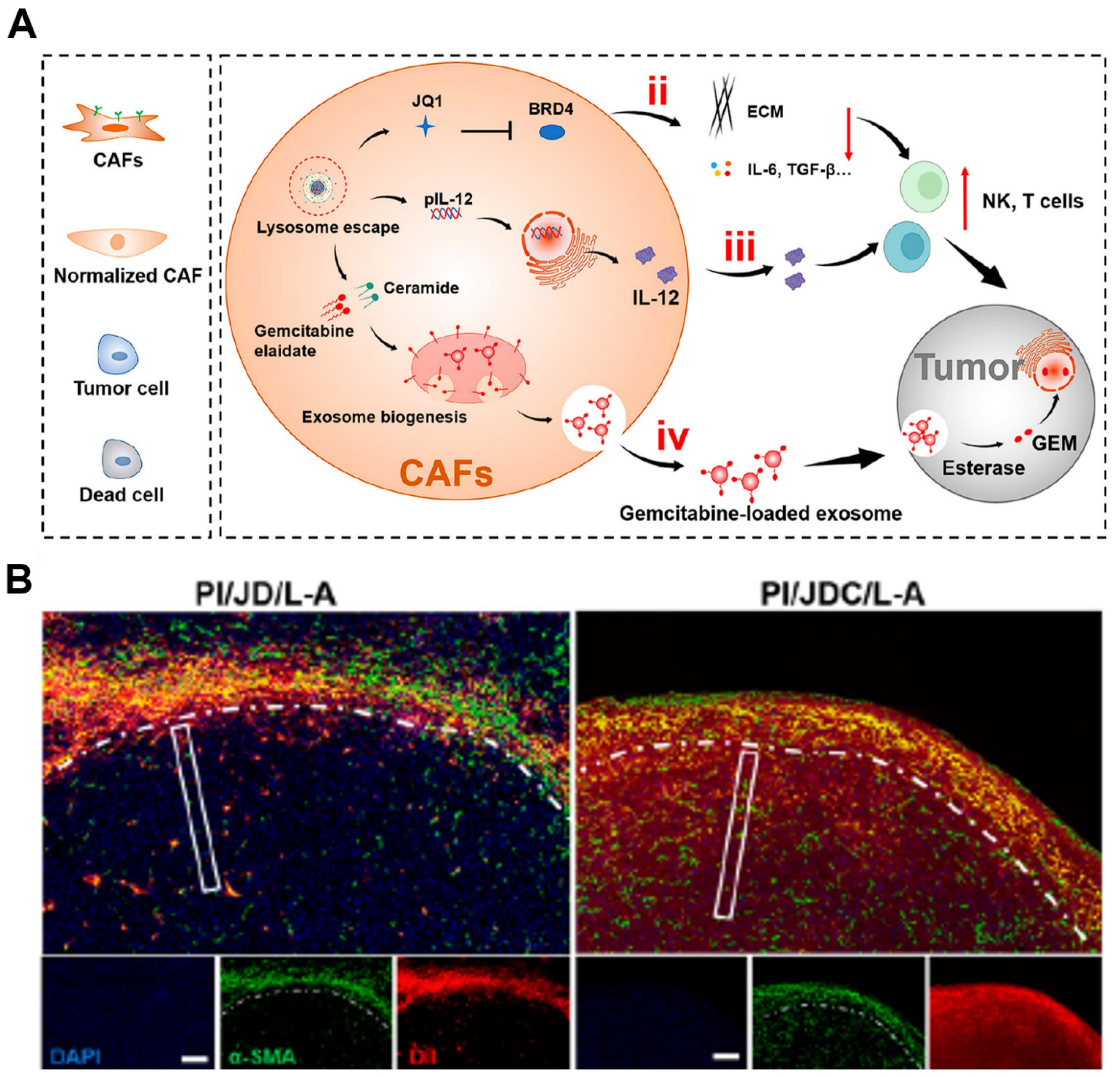
Engineering CAFs to turn enemies into friends. A) Schematic diagram of PI/JGC/L-A facilitating chemoimmunotherapy in a 'shooting fish in a barrel' model. B) Images of drug penetration depth in vivo by PI/JGC/L-A to cross through the CAF barrier. (A-B) Adapted with permission from 204, copyright 2023 American Chemical Society.

An emerging strategy involves reprogramming CAFs from stromal “enemies” into therapeutic “friends”. Yuan et al. recently advanced this concept by transforming the CAFs barrier into a drug reservoir, enabling chemo-immunotherapy in a “shooting fish in a barrel” fashion (**Figure 11A**) 204. In their work, a polymeric core (PI) with the encapsulation of plasmid-encoded IL-12 (pIL-12) and a liposomal shell (JGC/L-A) co-loaded with the epigenetic modulator JQ1 and the chemotherapeutic gemcitabine elaidate were integrated to generate PI/JGC/L-A nanosystem. Upon intratumoral administration, JQ1 and pIL-12 that were delivered in CAFs reprogrammed CAFs toward a quiescent phenotype, causing the reduction of ECM components and extracellular factors like IL-6, TGF-*β* and the increased production of IL-12, both of which facilitated immune-cell infiltration in tumors. Moreover, gemcitabine elaidate released in CAFs participated in exosome biogenesis, and the drug-loaded exosome spit out from CAFs further transformed into deep tumor site for efficient tumor cell killing. To this end, PI/JGC/L-A converted a formidable stromal barrier into self-powered therapeutic depots, simultaneously enhancing chemotherapy penetration and immunotherapy potency (**Figure 11B**), thereby establishing a rational foundation for turning CAF “foes” into functional allies.

It has been reported that abundant CAFs in malignant breast cancer could defunctionalize CD8 cytotoxic T-lymphocyte (CTL) directly, exacerbate T cell depletion and increase resistance to immune checkpoint blockade (ICB) therapy 205. Specially, CAFs possess antigen-processing capabilities similar to those of professional antigen-presenting cells (APCs) 206. Given that, Geng et al. reported a temperature-controlled gene expression nanosystem with the CD86 and PD-L1 trap plasmids (TNP@CS-A/plasmid (pDNA)) for engineering CAFs 29. Under laser-induced heat treatment, the HSP70-initiated plasmids, including CD86 and PD-L1 trap, were initiated to engineer immune-suppressed CAFs into immune-activated APCs, which could express co-stimulatory molecule (CD86) to activate antigen-specific CD8^+^ T cells (4-fold), and the PD-L1 trap protein secreted in situ was used for ICB therapy to avoid undesired immune cytokine storm. Through the strategy of turning enemies into friends, engineered CAFs may provide a promising paradigm for triggering anti-tumor immune response.

## 5. Current challenges and future prospects

Over recent years, rapid investigations of CAFs have revealed their critical involvement in cancer therapy, where they play a multifaceted role in tumorigenesis, progression, metastasis, and response to cancer therapy 109. This has triggered a wave of intellectual exploration aimed at designing strategies to effectively target CAFs, a major aspect of which is the use of nanosystems to address the challenge of CAF-driven cancer progression. This review presents an overview of the principal strategies for targeting CAFs with nanosystems, demonstrating that the activity of CAFs can be effectively regulated by nanomaterials to achieve metabolic reprogramming and remodeling of the TME, enhance drug penetration and improve therapeutic efficacy.

Despite the great progress on CAF-targeting NPs toward the development of advanced biomaterials in cancer therapy, the translational potential of these nanotherapeutics should be evaluated in light of not only their inherent strengths but also limitations. Polymer-based systems offer tunable degradation and functionalization properties for responsive drug release within fibrous stroma, yet concerns regarding toxic degradation products and monomer aggregation necessitate further refinement in synthesis and biosafety 207, 208. Metal NPs provide unique optical, magnetic, and photothermal properties for CAFs modulation, but their long-term biodistribution and potential ion toxicity remain unresolved 209. Although non-metal inorganic NPs provide versatile multimodal imaging and therapy potential, variability in particle size and surface charge remains an obstacle to reproducible performance that future studies hope to smooth out 210, 211. Membrane-camouflaged nanoplatforms exhibit superior immune evasion and targeting capability, yet limitations in membrane isolation technology and source availability may impede scalable production 212-214. Protein-based carriers are highly biodegradable and minimally immunogenic, but their complex assembly and variable in vivo behavior underscore the need for standardized manufacturing and detailed pharmacokinetic profiling 215. At a strategic level, direct CAFs depletion can rapidly disrupt stromal barriers to drug delivery, but it risks eliminating tumor-restraining CAFs subpopulations and triggering adverse stromal reactions, emphasizing the need for higher selectivity 39. Importantly, excessive or non-selective elimination of CAFs may dismantle the dense ECM they produce, thereby lowering tissue stiffness and releasing physical constraints that confine malignant cells 216. It has been reported that genetic or pharmacologic depletion of FAP-expressing stromal cells in triple-negative breast cancer reduced ECM density but simultaneously elevated the fraction of circulating tumor cells and enhanced seeding of distant organs 217. These findings underscore that, although the excessive activation of fibroblasts can hinder drug delivery, complete removal of the CAF-induced stromal barrier removes a critical “cage” that restrains tumor cells. Therefore, therapeutic strategies may favor CAFs reprogramming or subtype-specific targeting over broad ablation to preserve the restraining functions of CAFs and prevent inadvertent metastatic dissemination 218. Alternatively, interfering with CAF-activating cytokines may avoid wholesale depletion. However, the marked heterogeneity of CAFs and the pleiotropic roles of mediators such as TGF-*β* may render single-pathway inhibition insufficient or systemically disruptive, suggesting that multimodal synergistic therapy or sequential regimens could become essential 219. Reprogramming rather than removing CAFs is conceptually attractive, but current grasp of CAFs subpopulation dynamics remains limited, and nano-enabled gene editing tools are still in their infancy, indicating that further functional studies and delivery refinements will be indispensable. Only by confronting and systematically addressing these shortcomings can we hope to ultimately translate the encouraging preclinical findings into durable and reproducible patient benefit.

Despite the encouraging outcomes observed in representative studies, the clinical application of nanomaterial-based CAF-targeting strategies remains challenging. It is therefore instructive to explore the reasons behind the limited number of CAF-directed nanoplatforms that have reached clinical application. In the phase-Ib/II SWOG S1313 study (NCT01839487) and the phase-III HALO-301 trial (NCT02753595), the pegylated hyaluronidase PEGPH20 was added to standard nab-paclitaxel/gemcitabine with the aim of dismantling the CAF-secreted hyaluronan network that elevated interstitial pressure, collapsed tumor vessels and blocked drug and immune-cell entry. Despite robust evidence that hyaluronan depletion widened vessel caliber and improved gemcitabine delivery, the clinical study produced no survival benefit (HR 1.00, p = 0.97), highlighting the gap between CAF-rich murine stroma and human pancreatic tumors and how murine stroma may overestimate benefit and underestimate the risk of systemic hyaluronan loss 220, 221. Similarly, the Hedgehog-pathway inhibitor vismodegib (phase-II, NCT01255800) combined with gemcitabine produced no survival gain, possibly because complete Hedgehog blockade simultaneously removed tumor-restraining myofibroblasts and accelerated epithelial proliferation 222. In a Phase I dose-escalation study of sibrotuzumab in patients with FAP-positive advanced and metastatic solid tumors, the antibody rapidly and selectively accumulated within tumor stroma, confirming FAP-expressing CAFs as the pharmacologic target (NCT02209727). Despite this engagement, only transient disease stabilization was observed and 23% of subjects developed grade 3-4 thrombocytopenia, indicating that indiscriminate depletion of FAP-positive CAFs may compromise microvascular integrity and limit therapeutic efficacy 223. These failures reveal a central dilemma: CAFs assemble a stroma that simultaneously shields and constrains the tumor. Therapies that indiscriminately dismantle this barrier may unlock invasion and metastasis while offering no survival gain. Future nanomedicines may therefore silence only the pro-tumoral arm of CAFs biology while leaving their tumor-restraining functions. Achieving this selectivity will demand nanoplatforms that exploit unique surface signatures or metabolic phenotypes of CAFs, thereby silencing their tumor-promoting activity while leaving their restraining functions intact.

Moreover, the translational gap is widened by fundamental mismatches between pre-clinical models and human tumors: patient-derived CAFs cultures in 2D flatten into a myCAFs phenotype that rarely recapitulates the heterogeneous iCAFs/myCAFs admixture observed in resected pancreatic tumors, whereas 3D spheroids restore the immunosuppressive iCAFs signature and ECM architecture that dominate human lesions 224. Xenograft studies further rely on clonal FAP-over-expressing cell lines, resulting in uniform tracer uptake absent in patients where CAFs arise from multiple origins (resident fibroblasts, MSCs, adipocytes) and display variable or absent FAP expression, as highlighted by FAPI-PET heterogeneity across colorectal, ovarian and breast metastases 225. These discrepancies underscore why murine models overestimate CAF-targeting efficacy and underestimate systemic toxicity, necessitating validation in patient-derived organoids and spatially resolved human tissue analyses to better align nanomedicine design with clinical reality. Consequently, nanomedicine strategies should incorporate dynamic and responsive designs to adapt to CAFs plasticity. Firstly, future studies should pay more attention to the development of more accurate preclinical tumor models to make the results of in vitro simulation more convincing. On the other hand, given the partial phenotypic overlap between CAFs and normal fibroblasts, the biosafety and stability of these nanomaterials must be rigorously validated to ensure that they are safe for in vivo use. Although much is known about the physicochemical properties of NPs, their long-term biocompatibility, potential for immune system interactions, and overall safety still need to be thoroughly tested 207, 208. Moreover, determining the optimal dosing regimen (i.e., intravenous, oral or topical administration and frequency) and delivery method is critical to maximize the therapeutic potential of nanomedicines while minimizing side effects.

Additionally, in the complex biological systems formed by CAFs within tumors, the dense ECM and elevated interstitial fluid pressure form physical barriers that prevent effective drug delivery to the tumor site, which make it difficult to target and deliver nanomedicines to CAFs. Therefore, strategies to enhance NPs targeting, such as surface modifications with CAF-specific markers or receptors for selective recognition, will are highly needed to develop nanomedicines that can overcome the biological obstacles for elevating drug availability 196, 197. More importantly, identifying specific CAFs markers or receptors that can be targeted without affecting normal cells is critical to gain a deeper understanding of the molecular mechanisms of controlling CAFs function, which will enable researchers to design more effective nanomedicines to selectively target CAFs with minimal off-target side effects.

Another challenge lies in the production of nanomaterials, particularly because achieving targeting abilities or other functionalities for CAFs often requires complex modifications. Current methods for generating NPs that meet these performance standards typically produce heterogeneous populations with different sizes or structures, which can lead to inconsistent quality and the existence of impurities. This variability complicates their clinical application. To address this issue, more advanced techniques are needed to improve the quality, stability and purity of nanomaterials, especially during large-scale production. Standardized separation and purification processes with Good Manufacturing Practices (GMP) requirements and ensuring consistency and reproducibility are essential 226. However, GMP compliant methods for large-scale production of these nanomaterials are still lacking. Moreover, the development of multifunctional nanoplatforms in combination with immunotherapy and gene therapy is an exciting area of research. Such platforms can enhance the delivery of multiple therapeutic agents to CAFs and cancer cells to provide personalized treatment for individual patients. In addition, innovative delivery systems, such as nanoprobes and vaccines, could provide more precise targeting strategies to inhibit tumor growth through CAFs targeting. Nanomedicine should not be an isolated innovation but a complementary approach capable of addressing the pharmacokinetic limitations, poor selectivity, and safety issues associated with current CAF-directed therapies, thereby lowering clinical translation risks. By integrating these approaches, researchers can advance personalized medicine and pave the way for highly customized cancer therapies.

In summary, nanomedicines targeting CAFs have great potential to revolutionize cancer therapy by improving therapeutic efficacy through enhanced drug delivery and modulation of the TME. The ability of nanomaterials to specifically target CAFs and modulate their activity offers promising new approaches to address cancer progression, metastasis, and treatment resistance. However, there are also challenges to translating these advances from the laboratory to clinical practice. Future success will hinge on the integration of spatial multi-omics with digital pathology to map CAFs subpopulations in real time, enabling the development of nanocarriers capable of selectively reprogramming, rather than depleting, tumor-promoting subsets. With ongoing technological advances, CAF-targeted nanomedicine is likely to evolve from single-agent delivery systems into programmable platforms that can remodel the tumor stroma in concert with multimodal synergistic therapies. Such progress will translate precision stromal engineering into durable clinical benefits, ultimately improving therapeutic outcomes and quality of life for cancer patients.

## Figures and Tables

**Table 1:** Different nanomaterials for targeting CAFs.

NPs type	NPs name	Target Site	Mechanism	Therapeutic outcomes	Tumor model	Ref.
Polymers	MATT-LTSLs (Thermosensitive liposomes)	CAFs	Down-regulate TGF-*β* to block CAF activation, reduce MMP secretion, and preserve ECM integrity	Decrease tumor growth tenfold, inhibite metastasis completely, reduce angiogenesis; suppress CAF activation and ECM degradation	BRCA	22
	IRI-RGD/R9-sLip	FAP^+^ CAFs and tumor cells	Kill CAFs and tumor cells and promote drug penetration	Shrink tumor volume nearly 71%, down-regulate FAP and *α*-SMA expression, reduce tumor cell migration and lung metastasis	Colorectal cancer	134
	(PGA)/PolyMet-pRLN	CAFs	Metformin and relaxin co-loaded nanoparticles suppress CAF proliferation and ECM deposition, reduce TGF-*β* signaling, and increase T-cell infiltration	Inhibit tumor growth, reduce CAF content, increase CD8^+^ T cell infiltration and systemic immune response in triple-negative breast cancer (TNBC)	TNBC	135
	[OEGMA-Dendron(G2)-GFLG-DAS] and [OEGMA-Dendron(G2)-hydrazone-Epi]	CAFs	Dendritic polymer nanoparticles deplete CAFs via intracellular acid-triggered drug release, reduce collagen I deposition, reopen tumor vessels, and increase CD8^+^ T cell infiltration	Rebuild tumor stroma, double drug penetration, inhibit tumor growth and amplify systemic antitumor immunity	BRCA	138
	Hydrogel	CAFs	Sustain release losartan for 9 days, block TGF-*β* signaling, cut collagen synthesis, decompress vessels, boost DOX delivery	Shrink tumor growth 64%, wipe out lung metastasis 80%, lower CAF numbers and reduce collagen	BRCA	18
	PSN38@TPL-nsa (Polymeric micelles)	CAFs	Inactivate CAFs via NF-*κ*B and TGF-*β* blockade, reduce collagen/FAP/*α*-SMA, reopen vessels	Suppress primary tumor growth, abolish peritoneal metastasis, shrink CAF numbers, enhance irinotecan efficacy in gastric cancer	Gastric cancer	19
Metal nanoparticles	GNP	CAFs	Induce lipid synthesis, up-regulate *FASN*/*SREBP2*/*FABP3*, drive activated CAFs into lipid-rich quiescent state and cut collagen deposition	Revert activated CAFs to quiescence, reduce ECM production, sensitize CAFs to *FASN* inhibition, inhibit tumor growth in pancreatic cancer	PC	145
	AuNPs	Crosstalk between the pancreatic cancer cells and the pancreatic stellate cells	Trigger ER stress pathway, deplete TGF-*β*, PDGF, endostatin, MMP-9, drive PSCs to lipid-rich quiescence, cut collagen/*α*-SMA deposition	Reprogram tumor stroma, double vessel density, inhibit tumor growth, boost drug delivery and extend survival in orthotopic pancreatic cancer	PC	143
	PmMN@Om&As (Magnetic metal-organic framework)	CAFs and mitochondrial function of TILs	Release oxymatrine and astragaloside IV to inhibit CAF activation, reduce collagen/FAP/CXCL12, reopen vessels, increase TIL infiltration, boost TIL mitochondrial function	Suppress tumor growth 84%, prolong survival, shrink CAF numbers, expand CD8^+^ TILs, enhance systemic immunity in HCC	HCC	147
	Prune-to-essence nanoplatform (Pres) (superparamagnetic Fe_3_O_4_ NPs)	CAFs	Execute antiangiogenesis to rarefy vessels, eliminate CAFs to diminish collagen and loosen stiff structure, form positive feedback loop to deepen penetration, relieve immunosuppression, and induce immunogenic cell death	Regress tumor environment, inhibit primary and distant tumor growth, prolong survival, unleash systemic immune responses, and generate immune memory against TNBC	TNBC	148
	Au@Ag (core-shell NPs)	CAFs and tumor cells	Release silver ions, trigger transcriptome reprogramming, down-regulate metastasis-related secretome, arrest CAF cell cycle, break CAF-tumor cell crosstalk	Suppress metastasis, reduce lung metastatic mass and nodule number, weaken CAF pro-tumor activity, enhance DOX efficacy without systemic toxicity	Adenocarcinoma	142
Silica NPs	DMON-P (Dendritic mesoporous organosilica NPs)	CAFs	Release pioglitazone inside CAFs through GSH-responsive tetrasulfide-bridged dendritic mesoporous organosilica, activate PPAR-*γ*, suppress TGF-*β*/SMAD1/2, down-regulates* α*-SMA, Col1A1, vimentin and TGF-*β*, degrade collagen and normalize ECM	Reprogram CAFs into quiescent state, inhibit tumor growth, reduce metastasis, enhance DOX penetration and efficacy, decrease *α*-SMA and collagen in vivo	BRCA	151
Black phosphorus	Bioactive black phosphorus	iCAFs and myCAFs	Down-regulate TGF-*β*1 and RNA splicing of TGF-*β* pathway, block CAF differentiation trajectory, inhibit PDAC-CAF crosstalk	Reduce iCAF/myCAF numbers, deactivate CAFs in primary and liver metastatic PDAC, suppress metastasis, prolong survival	PDAC	154
Carbon dots	Carbon dots (CDs) Pep-APCDs@Fe/DOX-LOS	FAP-*α*^+^ CAFs	CAF-responsive peptide (FAP-*α*-sensitive) triggers disassembly of nanoassemblies, releasing LOS to degrade stromal collagen; DOX/ Fe ions induce ICD	84.8% tumor growth inhibition, complete prevention of lung metastasis, prolonged survival, enhanced infiltration of T cells and NK cells, reduced Tregs and MDSCs	BRCA	23
Cell membrane-based NPs	IEVs-PFD/138	CAFs	miR-138-5p inhibit FERMT2-TGFBR1 and FERMT2-PYCR1 complexes, suppress TGF-*β* signaling and proline-mediated collagen synthesis; PFD enhance anti-fibrotic effects	Reprogram CAFs, reduce ECM deposition, enhance gemcitabine penetration, inhibit tumor growth and metastasis, improve survival, decrease tumor pressure and hypoxia	PC	163
	FAP-CAR-CM@PLGA-AB NPs	FAP^+^ CAFs and senescent CAFs (SC CAFs)	Dual targeting via FAP scFV; ABT-263 clears senescent CAFs; nintedanib inhibits CAF activation and immunosuppressive cytokines (TGF-*β*, IL-6, IL-10)	Clear CAFs and SC CAFs; enhance radiotherapy efficacy; tumor inhibition rate up to 86.7%; reduce immunosuppression; increase CD8^+^ T cell infiltration; effective in radioresistant models	BRCA	169
	PTX/PFK15-SLN@[4T1-3T3] NPs	4T1 cancer cells and CAFs (3T3 fibroblasts activated by TGF-*β*1)	Dual homologous targeting via hybrid 4T1-3T3 membranes; PFK15 inhibits glycolysis in both cancer cells and CAFs, blocking energy supply and reducing lactate	Inhibit tumor, reduce lactate production, enhance T cell infiltration, decrease Tregs and M2 macrophages	BRCA	168
Protein-based NPs	Nutri-hijacker	KRAS-mutated pancreatic cancer cells	Trojan horse-like uptake via macropinocytosis; impairs glycolysis and inhibits glutaminolysis to induce synthetic lethality in mtKRAS cells	Suppress tumor cell proliferation and metastasis, reduce tumor fibrosis and immunosuppression, extend survival in combination with hydroxychloroquine	PDAC	173
	αFAP-Z@FRTs	FAP^+^ CAFs	PDT upon irradiation; generate ROS to selectively kill CAFs; elicit anti-cancer and anti-CAF immunity	Eliminate CAFs, reduces ECM, enhance CD8^+^ T cell infiltration, induce abscopal effect, synergize with anti-PD-1 therapy, retard distant tumor growth	4T1 breast cancer (mouse model), A549 lung cancer (adoptive transfer model)	176
	PDGFR-*β*-targeted self-assembling proteins	PDGFR-*β*^+^ CAFs	Selective CAFs destruction	Significant reduction in tumor volume and no systemic toxicity	Colorectal cancer	227

AuNPs: gold nanoparticles; BRCA: breast cancer; CAFs: cancer-associated fibroblasts; CAR: chimeric antigen receptor; CDs: carbon dots; ECM: extracellular matrix; Epi: epirubicin; FAP: fibroblast activation protein; *FASN*: fatty acid synthase; GNP: gold nanoparticles; HCC: hepatocellular carcinoma; ITGB1: integrin beta -1; MMP: matrix metalloproteinase; NPs: nanoparticles; NSCLC: non-small cell lung cancer; PC: pancreatic cancer; PDAC: pancreatic ductal adenocarcinoma; PDGFR-*β*: platelet-derived growth factor receptor beta; PPAR-*γ*: peroxisome proliferator-activated receptor gamma; PTX: paclitaxel; RLN: relaxin; SC-CAFs: senescent cancer-associated fibroblasts; TGF-*β*: transforming growth factor-beta; TILs: tumor-infiltrating lymphocytes; TME: tumor microenvironment; TNBC: triple-negative breast cancer.

**Table 2:** Nanomedicine strategies targeting CAFs in cancer therapy.

Therapeutic strategy	NPs name	Target Site	Mechanism	Therapeutic outcomes	Tumor model	ref.
Direct CAFs depletion	PNP-D-mAb	FAP^+^ CAFs and cancer cells	Anti-FAP mAb targeting CAFs, CPP exposure, enhance DOX penetration into CAFs and tumor cells	Decrease tumor growth; *α*-SMA^+^ CAFs nearly eliminated, 10-fold higher tumor accumulation	Prostate tumor	59
	Ca^2+^-doped PDA	CAFs and tumor cells	Trigger Ca^2+^ release to damage lysosomes, block autophagy flux, accumulate autophagosomes; induce glutamine starvation to enhance macropinocytosis; apply photothermal effect to reduce CAFs	Inhibit tumor growth by nearly 80%, reduce lung metastasis, degrade ECM, decrease CAFs; enhance ICD and generate immune memory	Pancreatic tumor	24
	FAP-MRI/PET probes	FAP^+^ CAFs and tumor cells	Dual-modal imaging and subtype-specific targeting for precision CAFs therapy	Achieve PET/MR/PA multimodal imaging, inhibit tumor growth to 21.3% of control, induce apoptosis and reduce CAFs and collagen	Multiple solid tumors	25
	FAP_PEP_-SLNPs	FAP^+^ CAFs	Display FAP immunodominant CD8^+^ and CD4^+^ T cell epitopes, activate DC cross-presentation and elicit FAP-specific CTL responses	Deplete FAP^+^ CAFs, reduce collagen I and fibronectin, increase CD8^+^ T cell infiltration, suppress tumor growth by nearly 70% and enhance anti-PD-1 efficacy	T-cell lymphoma	56
	E_8_R_8_-Lip	CAFs (FAP-*α*^+^ and *α*-SMA^+^) and tumor cell mitochondria	Deplete CAFs to reduce ECM and increase tumor oxygen perfusion, disrupt mitochondrial respiration to decrease oxygen consumption, downregulate hypoxia-induced factors (LOX, MMP2) and CAF-secreted cytokines (CXCL12, IL-6, TGF-*β*), and inhibit CAF-tumor cell crosstalk	Alleviate tumor hypoxia, inhibit primary tumor growth, prevent pre-metastatic niche formation in lung, suppress cancer cell migration and invasion, eradicate lung metastasis, and exhibit good biocompatibility	BRCA	186
	Oxygen-delivering polyfluorocarbon nanosystem loading DiIC18 and halofuginone (M-FDH)	CAFs and ECM	Selecte DiD as a radio-sensitizer to enhance the production of free radicals upon X ray irradiation and use halofuginone (HF) (M-FDH) to improve intratumor delivery and relieve tumor hypoxia	Eliminate over 90% of CAFs and major ECM, boost infiltration and function of CD8^+^ T cells, reduce immunosuppressive cells (M2 macrophages, MDSCs, Tregs), inhibit tumor growth in multiple models, and synergize with *α*PD-L1 therapy	BRCA	188
	F-SOS/DC NC	FAP^+^ CAFs	Target CAFs via FAP-*α*, use light irradiation to generate singlet oxygen which concurrently depletes CAFs and degrades the NCs' shell, release small-sized and positively charged DC/D for deep penetration, and synergize DOX chemotherapy with Ce6 photodynamic therapy	Mediate hierarchical intratumoral penetration and programm antitumor therapy, eradicate tumor cells deep in solid tumors through cooperative chemo-photodynamic therapy, and strengthen anticancer efficacy in CAF-rich tumors by remodeling the TME	Colorectal cancer	179
	FH-SSL-Nav	CAFs	Target and deplete CAFs via FH peptide, disrupt collagen barrier, and promote penetration of chemotherapeutic NPs	Synergize anti-tumor efficacy, reverse drug resistance, and enhance chemotherapy effect	Hepatocellular carcinoma (HCC)	181
Block CAFs' biological function for TME remodeling	JQ1&PFD@CTL	CAFs	Use CAF membrane for homologous targeting to deliver PFD and BETi, disrupt fibrous matrix, improve tumor hypoxia, suppress glycolysis, and enhance chemosensitivity	Enhance drug accumulation in tumor, disrupt stromal barrier, improve chemotherapy efficacy, and modulate tumor metabolism in pancreatic cancer	Pancreatic ductal adenocarcinoma	228
	Gemini-like NPs (siNNMT-MSN@CAFs membrane)	CAFs	Deliver si*NNMT* to CAFs to reprogram vitamin B3 metabolism and epigenetic profile, reducing pro-tumorigenic secretome and restoring chemosensitivity	Reverse chemoresistance, reduce cancer stem cells and immunosuppressive cells, enhance CD8^+^ T cell infiltration, achieve complete tumor regression and generate long-term immune memory in multiple tumor models	BRCA	201
	DAS@CDC	CAFs and tumor cells	Reprogram CAFs to a normal phenotype via dasatinib, reduce ECM secretion, block tumor-stroma crosstalk, and synergize with cabazitaxel to kill tumor cells	Inhibit primary tumor growth, prevent lung metastasis, reverse EMT, downregulate ECM-related genes, and exhibit outstanding biosafety in breast cancer	BRCA	20
	FPC@S	CAFs and ECM	Target fibronectin in ECM, use PDT to degrade ECM and induce immunogenic cell death, release SIS3 to reprogram CAFs and reduce ECM production, alleviate tumor hypoxia	Reshape ECM and reprogram CAFs to enhance drug penetration and immune cell infiltration, augment PDT, inhibit primary tumor growth and metastasis, and synergize with *α*PD-L1 for robust immunotherapy	BRCA	229
	Carrier-free nanoagent (CFNA)	CAFs	Inhibit ACLY in CAFs to disrupt lipid metabolism, reduce ATP and ECM (collagen, fibronectin) production, and enhance chemotherapeutic drug penetration	Enhance deep tumor drug delivery, achieve high-performance chemotherapy, and produce synergistic tumor killing in OSCC	OSCC	196
	PMNPs-D	CAFs	Use FAP-*α*-cleavable peptide to site-specifically release FNIII14 peptide, which inactivates ITGB1 in CAFs to inhibit their profibrotic function and reduce ECM stiffness	Loosen ECM structure, enhance deep tumor drug penetration, boost chemotherapeutic efficacy, and achieve high-performance tumor cell killing in ACC	Adenoid cystic carcinoma	197
	NS@Lipo-VAC	CAFs and cancer cells	Target fibronectin for tumor retention, release VAC to deactivate PSCs and reduce ECM, and release small NS-TAX for deep penetration, creating a cascaded drug delivery loop	Effectively suppress tumor progression in a desmoplastic PDAC model by overcoming the stromal barrier and enabling deep drug delivery	PDAC	195
	The Epi-PEGylated dendron prodrug (Epi-PD) with 4 PEG molecules	CAFs and tumor cells	Disturb amino acid metabolism in CAFs to reduce ECM deposition, promote deep tumor penetration, and release epirubicin to induce immunogenic cell death	Achieve superior tumor penetration and nearly 80% tumor growth inhibition, boost antitumor immune cell infiltration (NK, CD4^+^, CD8^+^ T cells), and reduce systemic toxicity	Colon cancer and BRCA	28
Engineering modifications to CAFs	PI/JGC/L-A	CAFs	Normalize CAFs (rather than eliminate) with JQ1 to reduce ECM, use CAFs as factories to produce IL-12 and gemcitabine-loaded exosomes for deep tumor delivery	Inhibit PDAC growth, reverse immunosuppressive TME, promote immune cell infiltration, and prolong survival in orthotopic models	PDAC	204
	sTRAIL	CAFs	Utilize natural off-target uptake by CAFs to deliver sTRAIL plasmid, engineer CAFs to secrete sTRAIL protein which induces apoptosis in neighboring tumor cells, and subsequently reprogram residual CAFs to a quiescent state	Induce potent tumor inhibition, remodel the tumor microenvironment, and create a window for second-wave nanoparticle therapy in desmoplastic tumors	PC	27
	TNP@CS-A/plasmid (pDNA)	CAFs engineer as APCs via co-stimulatory molecule (CD86) expression	Utilize precise photothermal control to safely transfect CAFs, engineer them into antigen-presenting cells (APCs) that express CD86 and secrete PD-L1 trap protein	Transform CAFs from foes to friends, activate and proliferate CD8^+^ T cells, block PD-1/PD-L1 pathway in situ without immune storm, and improve immunotherapy in fibrotic breast cancer	Highly fibrotic BRCA	29
	FAP-Ad5	FAP^+^ CAFs	Use FAP-specific adapter to retarget adenovirus to CAFs, engineer them as local “biofactories” to produce and secrete anti-cancer therapeutics in the TME	Enable stromal cell-targeted delivery of therapeutic payloads, reduce tumor growth, and provide a platform for local production of biologics in the TME	Gastric cancer	230

APCs: antigen-presenting cells; BRCA: breast cancer; BRD4: bromodomain-containing protein 4; CAFs: cancer-associated fibroblasts; CFNA: carrier-free nanoagent; CTL: cytotoxic t lymphocytes; DAS: dasatinib; ECM: extracellular matrix; FAP: fibroblast activation protein; HCC: hepatocellular carcinoma; JQ1: bromodomain inhibitor; MRI: magnetic resonance imaging; *NNMT*: nicotinamide n-methyltransferase; OSCC: oral squamous cell carcinoma; PDA: polydopamine; PDAC: pancreatic ductal adenocarcinoma; PDGFR: platelet-derived growth factor receptor; PET: positron emission tomography; PFD: pirfenidone; TAFs: tumor-associated fibroblasts; TGF-*β*1: transforming growth factor beta 1; TME: tumor microenvironment; VB3: vitamin b3.

## Abbreviations

AEAA: aminoethyl anisamide
apCAFs: antigen-presenting cancer-associated fibroblasts
ATP: adenosine triphosphate
APCs: antigen-presenting cells
As: astragaloside IV
*α*-SMA: *α*-smooth muscle actin
BMSCs: bone marrow mesenchymal stem cells
BP: black phosphorus
CAFs: cancer-associated fibroblasts
CCL2: c-c motif chemokine ligand 2
CDs: carbon dots
CSC: cancer stem cells
CTL: cytotoxic T-lymphocyte
DAS: dasatinib
DOX: doxorubicin
ECM: extracellular matrix
EMT: endothelial-to-mesenchymal transition
Epi: epirubicin
EPR: enhanced permeability and retention
EVs: extracellular vesicles
FAK: focal adhesion kinase
FAP: fibroblast activation protein
FAPI: fibroblast activation protein inhibitor
*FASN*: fatty acid synthase
*GAS1*: growth arrest specific 1
GMP: good manufacturing practices
GNPs: gold nanoparticles
GSH: glutathione
HIF-1*α*: hypoxia-inducible factor-1*α*
HSP70: heat shock protein 70
iCAFs: inflammatory cancer-associated fibroblasts
ICB: immune checkpoint blockade
ICD: immunogenic cell death
IL-6: interleukin 6
IRE1: inositol-requiring enzyme 1
ITGB1: integrin *β*1
LND: lonidamine
LOS: losartan
LTSLs: lysolipid thermosensitive liposomes
mAb: monoclonal antibody
MATT: marimastat
myCAFs: myofibroblast cancer-associated fibroblasts
MHC I: major histocompatibility complex class I
MOFs: metal-organic frameworks
MSN: mesoporous silica nanoparticle
mtKRAS: kirsten rat sarcoma viral oncogene homolog mutations
NF-*κ*B: nuclear factor kappa-light-chain-enhancer of activated B cells
*NNMT*: nicotinamide n-methyltransferase
NPs: nanoparticles
Om: oxymatrine
PDA: polydopamine
PDGF: platelet-derived growth factor
PDGFR-*β*: platelet-derived growth factor receptor-*β*
PD-L1: programmed cell death protein 1
PDT: photodynamic therapy
PEG: polyethylene glycol
PET: positron emission tomography
PFD: pirfenidone
Pio: pioglitazone
Pm: platelet membrane
PPAR-*γ*: peroxisome proliferator-activated receptor-*γ*
PSCs: pancreatic stellate cells
Ptn: pleiotrophin
PTT: photothermal therapy
PTX: paclitaxel
RNA-seq: RNA sequencing
ROS: reactive oxygen species
SAM: s-adenosyl methionine
sCAFs: senescent cancer-associated fibroblasts
scFv: single-chain fragment variable
Smad: drosophila mothers against decapentaplegic
SPARC: secreted protein acidic and rich in cysteine
*Sp1*: specificity protein 1
Spp1: secreted phosphoprotein 1
SREBP2: sterol regulatory element-binding protein 2
STAT3: signal transducer and activator of transcription 3
Su: sunitinib
TGF-*β*: transforming growth factor *β*
TILs: tumor-infiltrating T lymphocytes
TME: tumor microenvironment
VAC: vactosertib
VEGF: vascular endothelial growth factor
*YAP*: yes-associated protein
